# Novel formylpeptide receptor 1/2 agonist limits hypertension-induced cardiovascular damage

**DOI:** 10.1093/cvr/cvae103

**Published:** 2024-06-16

**Authors:** Jaideep Singh, Kristy L Jackson, Haoyun Fang, Audrey Gumanti, Bethany Claridge, Feng Shii Tang, Helen Kiriazis, Ekaterina Salimova, Alex M Parker, Cameron Nowell, Owen L Woodman, David W Greening, Rebecca H Ritchie, Geoffrey A Head, Cheng Xue Qin

**Affiliations:** Drug Discovery Biology, Monash Institute of Pharmaceutical Sciences, Monash University, 381 Royal Parade, Parkville, VIC 3052, Australia; Baker Heart & Diabetes Institute, 75 Commercial Rd, Melbourne, VIC 3004, Australia; Drug Discovery Biology, Monash Institute of Pharmaceutical Sciences, Monash University, 381 Royal Parade, Parkville, VIC 3052, Australia; Baker Heart & Diabetes Institute, 75 Commercial Rd, Melbourne, VIC 3004, Australia; Baker Heart & Diabetes Institute, 75 Commercial Rd, Melbourne, VIC 3004, Australia; Department of Cardiometabolic Health, University of Melbourne, Melbourne, VIC, Australia; Drug Discovery Biology, Monash Institute of Pharmaceutical Sciences, Monash University, 381 Royal Parade, Parkville, VIC 3052, Australia; Baker Heart & Diabetes Institute, 75 Commercial Rd, Melbourne, VIC 3004, Australia; Baker Heart & Diabetes Institute, 75 Commercial Rd, Melbourne, VIC 3004, Australia; Drug Discovery Biology, Monash Institute of Pharmaceutical Sciences, Monash University, 381 Royal Parade, Parkville, VIC 3052, Australia; Baker Heart & Diabetes Institute, 75 Commercial Rd, Melbourne, VIC 3004, Australia; Department of Cardiometabolic Health, University of Melbourne, Melbourne, VIC, Australia; Monash Biomedical Imaging, Monash University, Clayton, Melbourne, VIC, Australia; Drug Discovery Biology, Monash Institute of Pharmaceutical Sciences, Monash University, 381 Royal Parade, Parkville, VIC 3052, Australia; Drug Discovery Biology, Monash Institute of Pharmaceutical Sciences, Monash University, 381 Royal Parade, Parkville, VIC 3052, Australia; Drug Discovery Biology, Monash Institute of Pharmaceutical Sciences, Monash University, 381 Royal Parade, Parkville, VIC 3052, Australia; Baker Heart & Diabetes Institute, 75 Commercial Rd, Melbourne, VIC 3004, Australia; Department of Cardiometabolic Health, University of Melbourne, Melbourne, VIC, Australia; Central Clinical School, Monash University, Melbourne, VIC, Australia; Department of Cardiovascular Research, Translation and Implementation, La Trobe University, Melbourne, VIC, Australia; Drug Discovery Biology, Monash Institute of Pharmaceutical Sciences, Monash University, 381 Royal Parade, Parkville, VIC 3052, Australia; Baker Heart & Diabetes Institute, 75 Commercial Rd, Melbourne, VIC 3004, Australia; Department of Cardiometabolic Health, University of Melbourne, Melbourne, VIC, Australia; Baker Heart & Diabetes Institute, 75 Commercial Rd, Melbourne, VIC 3004, Australia; Drug Discovery Biology, Monash Institute of Pharmaceutical Sciences, Monash University, 381 Royal Parade, Parkville, VIC 3052, Australia; Baker Heart & Diabetes Institute, 75 Commercial Rd, Melbourne, VIC 3004, Australia; Department of Pharmacology, School of Pharmaceutical Sciences, Qilu College of Medicine, Shandong University, 44 Wenhua Xilu, Jinan, Shandong 250012, PR China; Department of Emergency Medicine, Qilu Hospital of Shandong University, 107 Wenhua Xilu, Jinan, Shandong 250012, PR China

**Keywords:** Formylpeptide receptors, Compound17b, Angiotensin II, Hypertension, End-organ damage, Proteomics

## Abstract

**Aims:**

Formylpeptide receptors (FPRs) play a critical role in the regulation of inflammation, an important driver of hypertension-induced end-organ damage. We have previously reported that the biased FPR small-molecule agonist, compound17b (Cmpd17b), is cardioprotective against acute, severe inflammatory insults. Here, we reveal the first compelling evidence of the therapeutic potential of this novel FPR agonist against a longer-term, sustained inflammatory insult, i.e. hypertension-induced end-organ damage. The parallels between the murine and human hypertensive proteome were also investigated.

**Methods and results:**

The hypertensive response to angiotensin II (Ang II, 0.7 mg/kg/day, s.c.) was attenuated by Cmpd17b (50 mg/kg/day, i.p.). Impairments in cardiac and vascular function assessed via echocardiography were improved by Cmpd17b in hypertensive mice. This functional improvement was accompanied by reduced cardiac and aortic fibrosis and vascular calcification. Cmpd17b also attenuated Ang II-induced increased cardiac mitochondrial complex 2 respiration. Proteomic profiling of cardiac and aortic tissues and cells, using label-free nano-liquid chromatography with high-sensitivity mass spectrometry, detected and quantified ∼6000 proteins. We report hypertension-impacted protein clusters associated with dysregulation of inflammatory, mitochondrial, and calcium responses, as well as modified networks associated with cardiovascular remodelling, contractility, and structural/cytoskeletal organization. Cmpd17b attenuated hypertension-induced dysregulation of multiple proteins in mice, and of these, ∼110 proteins were identified as similarly dysregulated in humans suffering from adverse aortic remodelling and cardiac hypertrophy.

**Conclusion:**

We have demonstrated, for the first time, that the FPR agonist Cmpd17b powerfully limits hypertension-induced end-organ damage, consistent with proteome networks, supporting development of pro-resolution FPR-based therapeutics for treatment of systemic hypertension complications.

## Graphical Abstract

**Graphical Abstract:**
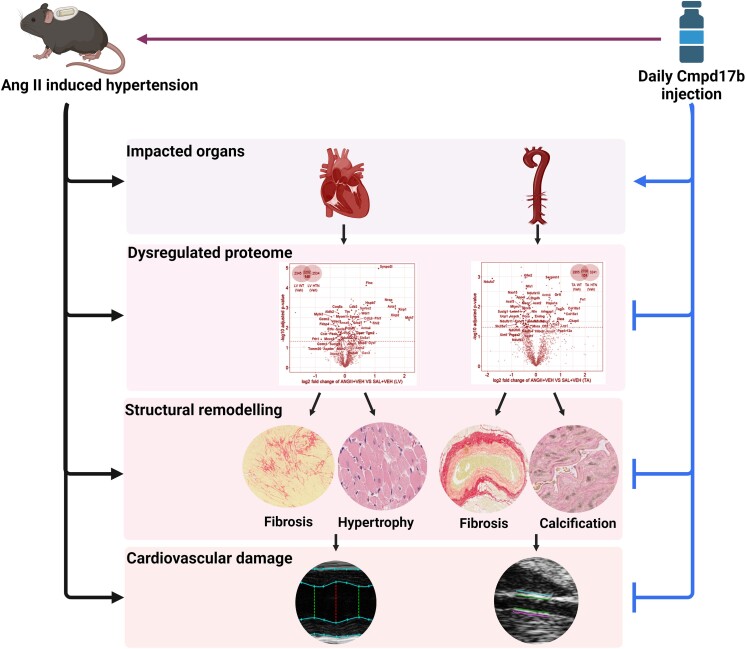

**Time of primary review: 56 days**

## 1. Introduction

Damage in the heart, kidney, and blood vessels is a pathological feature of hypertension, responsible for significant morbidity and mortality.^1^ Current antihypertensive therapies have limited efficacy in treating hypertension-induced end-organ damage.^2^ Further, clinical reports indicate that asymptomatic complications, such as left ventricular (LV) hypertrophy and aortic aneurysm,^3^ are also evident in patients even with well-managed hypertension. These observations highlight that current strategies may only attenuate the blood pressure elevation, without targeting underlying mechanisms of hypertension, such as unresolved inflammation, which contribute to end-organ injury.^4^

Research over the past decade has provided evidence of the contribution of inflammation to the development of hypertension and end-organ damage.^5^ Hypertension represents a chronic, low-grade inflammatory insult, which contributes to adverse structural remodelling and mitochondrial dysfunction, leading to cardiovascular complications.^6^ Extensive attention has focused on suppressing inflammation, e.g. via non-steroidal anti-inflammatory drugs (NSAIDs).^7^ However, elevation of blood pressure and sodium retention has been observed in patients treated with NSAIDs.^5,7^ An alternative approach may promote an active, beneficial processes for the resolution of inflammation to prevent or reverse adverse structural and mitochondrial remodelling.^8^

It is well-recognized that a failure to resolve inflammation may contribute to hypertension-induced end-organ damage.^5^ The formylpeptide receptor (FPR) family, a group of G protein–coupled receptors with three known subtypes in humans (FPR1, FPR2, and FPR3), plays a critical role in the regulation of the resolution of inflammation.^9^ FPRs have been regarded as predominantly located on monocytes and neutrophils,^9^ but their expression has more recently been reported on cardiomyocytes, cardiac fibroblasts, and vascular smooth muscle cells.^10,11^ Previous studies reported reduced expression of the endogenous FPR1/FPR2 agonist, annexin-A1 (AnxA1^12^) in hypertensive rodents, likely contributing to adverse vascular remodelling.^13^ We have demonstrated that the administration of the AnxA1 N-terminal peptide annexin 1-(2-26) blunted myocardial infarction (MI)-induced cardiac fibrosis, indicative of cardioprotection in response to a severe inflammatory insult.^14^ The specialized pro-resolving mediator (SPM) resolvin D2 attenuates the long-term inflammatory insult associated with hypertension, with the enhanced resolution of inflammation implicated in the attenuation of cardiovascular damage.^15^

The impact of FPR activation and the accompanying influence on regulation of inflammation and its resolution on hypertension-induced cardiac and vascular damage have not been reported. Given the limitations of endogenous FPR agonists such as AnxA1 (including short half-life and instability), pro-resolving small-molecule FPR agonists are particularly attractive for translation.^16^ The FPR2 agonist BMS-986235 progressed to early clinical trial (NCT03335553), confirming its safety in healthy subjects.^16^ We identified a small-molecule biased FPR1/FPR2 agonist, compound17b (Cmpd17b), that favours beneficial, pro-survival signalling pathways (e.g. FPR1/FPR2-mediated ERK/Akt activation) over detrimental Ca^2+^ mobilization, to exhibit superior cardioprotection compared to the non-biased FPR1/FPR2 agonist compound 43.^11^ Cmpd17b is reported to attenuate MI-induced LV neutrophil infiltration, inflammation, and contractile dysfunction in mice,^11^ confirming its cardioprotective activity. Further, Cmpd17b preserves endothelial function in mice, in part by up-regulation of vasodilator prostanoids including prostacyclin.^10^ Taken together, the cardio- and vasoprotective effects of Cmpd17b support investigation of the potential for this FPR agonist to limit hypertension-induced end-organ damage. Hence, the objective of this study was to examine, for the first time, the effect of the FPR agonist Cmpd17b on blood pressure, cardiovascular function and remodelling, and their underlying mechanisms, in hypertensive mice. We incorporated comprehensive comparative analysis of the mouse cardiac and aortic proteome and investigated its parallels to the human cardiac and aortic proteome, to gain insights into the mechanism(s) of action of Cmpd17b.

## 2. Methods

### 2.1 Animals and experimental design

This study was carried out in accordance with the recommendations of the Australian code for the care and use of animals for scientific purposes, National Health and Medical Research Council. The protocol was approved by the Alfred Medical Research Education Precinct Animal Ethics Committee (E/1987/2020/B) and was conducted in accordance with Animal Research: Reporting of In Vivo Experiments (ARRIVE) guidelines. Male C57BL/6J mice (*n* = 47) at 12 weeks of age were divided into four groups: vehicle-treated normotensive mice (saline-infused vehicle-treated mice), Cmpd17b-treated normotensive mice (saline-infused mice treated with Cmpd17b), vehicle-treated hypertensive mice [angiotensin II (Ang II)-infused vehicle-treated mice)], and Cmpd17b-treated hypertensive mice (Ang II-infused mice treated with Cmpd17b) and implanted with telemetry probes, under isoflurane open circuit anaesthesia (4% induction and 1.5–2% maintenance; Forthane, Abbott, Botany, Australia) to measure arterial pressure and locomotor activity. Hypertension was induced by subcutaneous infusion of Ang II at a dose of 0.7 mg/kg/day for 28 days as described previously.^17^ Cmpd17b (50 mg/kg/day) or vehicle (10% DMSO in 0.8% Tween80 with saline) was administered intraperitoneally once daily at ∼9:00 am for 28 days concurrent with Ang II infusion. Power spectral analysis was performed using LabView with long-term blood pressure to measure sympathetic activity. Cardiac and vascular function was assessed by ultrasound imaging using a Vevo 2100 High-Resolution Imaging System (Visual Sonics Inc., Canada). Oxygen consumption rates (OCRs) were measured in homogenates from frozen LV tissues of mice. Animals were euthanized with pentobarbital sodium (100 mg/kg, i.p.). Histological analysis of the heart, kidney, and aorta was quantified using automated and semi-automated macros in ImageJ software (version 1.53K14, National Institute of Health, USA). A flowchart of animal use and analysis is included based on the CONSAERT template (see Supplementary material online, *Figure S1*). Detailed methods are available in Supplementary material.

### 2.2 Quantitative proteomic analysis

Quantitative proteomic analysis of mouse left ventricle (LV, 5 mg) and thoracic aorta (TA, 2 mg) was performed, with stringent informatic analyses and correlation with adverse aortic remodelling^18^ and hypertrophic^19^ human data. To maintain relevance specifically to hypertension, we have selected the human studies of Herrington *et al*.^18^ and Coats *et al*.^19^ to align with our mouse and human cell proteome. Herrington *et al*. included male hypertensive patients, whereas Coats *et al*. included patients with aortic remodelling, both consistent with the male mouse in the current study. Briefly, defined Gene Ontology (GO) biological process and Kyoto Encyclopedia of Genes and Genomes (KEGG) pathway analysis enrichment were performed for each network using a hypergeometric distribution with Benjamini–Hochberg FDR correction for *P*-value calculation, enabling distinction of processes unique to disease or treatment groups.^20^ Detailed methods are available in Supplementary material.

### 2.3 Cell culture

Human aortic smooth muscle cells (HASMCs) were obtained from the American Type Culture Collection (ATCC Manassas, VA, USA),^21^ and cardiac fibroblasts (HCFs) were obtained from Lonza (CC-2904, batch 20TL356511; ventricle).^22^ HASMCs were cultured in basal vascular cell media containing 5% foetal bovine serum (FBS) in a humidified incubator at 37°C with 5% CO_2_. Cells from Passages 5–8 were used for the gene expression and proteomic analysis. Briefly, HASMCs were exposed to Ang II (300 nM) for 6 h in the presence of Cmpd17b (10 μM) or vehicle (0.1% DMSO). At the end of 6 h, cells were collected, and RNA was extracted for gene expression analysis. In parallel, cells were lysed for proteomics analysis, 24 h post Ang II stimulation.

HCFs were cultured in media consisting of 50% cardiac fibroblast growth medium, 45% DMEM/F12, 5% 0.22 μm filtered foetal calf serum, and penicillin–streptomycin at 37°C with 5% CO_2_. In brief, HCFs were stimulated with Ang II (1 μM) in the presence of Cmpd17b (10 μM) or vehicle (0.1% DMSO) for 24 h. Subsequently, the cells from Passage 6 were lysed for proteomic analysis as described in Supplementary material.

### 2.4 Statistical analysis

All analyses were performed with GraphPad Prism 9.0.1 (GraphPad, San Diego, CA). All the data were presented as mean ± SEM (*n* = 5–12). The arterial pressure data were analysed by a two-factor split-plot analysis of variance (ANOVA), which allowed for between-animal contrasts. Factors and interactions were deemed significant when *P* < 0.05 after adjustment by the Greenhouse–Geisser co-efficient. For gene expression, statistical analysis was performed using a one-way ANOVA followed by Bonferroni significant difference *post hoc* test. The rest of the data were analysed using two-way ANOVA followed by the Bonferroni *post hoc* test for significance. A *P* < 0.05 was considered significant.

## 3. Results

In the present study utilizing the Ang II-induced mouse model of hypertension, we confirmed that mean arterial pressure (MAP, determined via telemetry at 10 timepoints over 4 weeks) was higher in all mice infused with Ang II compared with mice infused with saline for 28 days (*P* < 0.001). MAP was not different in normotensive mice treated with vehicle or Cmpd17b (*P* > 0.05, Supplementary material online, *Table S1*).

### 3.1 Impact of Ang II-induced hypertension on the cardiovascular proteome

**Figure 1:**
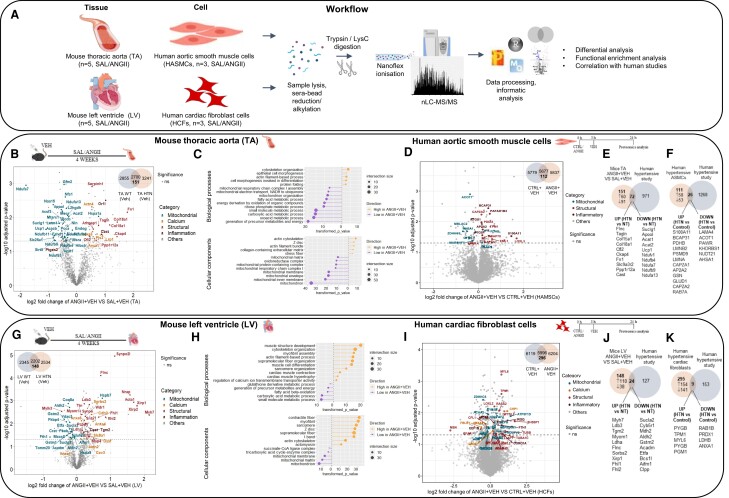
Sustained Ang II up-regulated cardiac and vascular structural, inflammatory, and calcium regulatory proteins and down-regulated mitochondrial proteins. Proteomic workflow (*A*) in both mouse tissues and human cell line, with corresponding results illustrated in (*B*–*I*). Volcano plots (with corresponding Venn diagram in the inset) displaying proteins identified in our study in mice (*B* and *G*) and human cell lines (*D* and *I*) with structural (red), calcium regulatory (orange), inflammatory (brown), and mitochondrial (blue) proteins. Up-regulated (yellow) and down-regulated (purple) biological processes (top lollipop panel) and cellular components (bottom lollipop panel) in TA (*C*) and LV (*H*). Venn diagrams display commonly identified up- and down-regulated proteins from our study and published human proteomic data set (*E–K*). Venn diagram comparing the TA mouse proteome from this study with the TA proteome of human aortic remodelling data set from Herrington *et al.*^18^ (*E*). Venn diagram compared the HASMC proteome from this study with the human aortic remodelling TA proteome (*F*). Venn diagram compared the LV mouse proteome from this study with the LV proteome of human hypertensive data set from Coats *et al.*^19^ (*J*). Venn diagram compared the HCF proteome from this study with the human hypertensive LV proteome (*K*). GO, gene annotation; BP, biological process; CC, cellular component; HTN, hypertensive; NT, normotensive; Veh, vehicle; Ang II, angiotensin II; Ctrl, control; Sal + Veh, vehicle-treated normotensive mice; AngII + Veh, vehicle-treated hypertensive mice; HCFs, human cardiac fibroblasts; HASMCs, human aortic smooth muscle cells.

A detailed workflow of mouse aorta (TA) and LV and HASMC and HCF proteomics based on sequential extraction of tissue (*n* = 5 per group) and cells (*n* = 3), respectively, followed by quantitative label-free tandem mass spectrometry and stringent informatic analyses, quantified ∼6000 proteins (*Figure 1A, Schema was created in BioRender*). A total of 3241 and 2534 proteins were identified in TA (see Supplementary material online, *Table S11*) and LV (see Supplementary material online, *Table S12*), respectively, with 2729 TA proteins and 2239 LV proteins commonly identified across all groups. Out of these commonly identified proteins, 151 TA and 148 LV proteins were different in hypertensive mice compared with normotensive mice (*P* < 0.05, *Figure 1B* and *G*, Supplementary material online, *Tables S13* and *S15*, *S19*). To complement our vascular remodelling data demonstrating dysregulated collagen and elastin deposition, predominantly in the aortic smooth muscle layer, a proteomic analysis in HASMCs was performed. In addition, given that greater expression of both FPR1 and FPR2 in cardiac fibroblasts than in cardiomyocytes has been reported, we also performed a proteomic analysis in HCFs.^14^ A total of 5677 and 5996 proteins were commonly identified in HASMCs and HCFs (*P* < 0.05, *Figure 1D* and *I*, Supplementary material online, *Tables S20*, *S22*, *S24*, and *S25*), respectively. Out of these commonly identified proteins, 112 HASMC and 296 HCF proteins were different in Ang II-treated cells compared with saline-treated cells (*P* < 0.05, *Figure 1D* and *I*, Supplementary material online, *Tables S20* and *S22*).

We first compared the mouse TA proteome of hypertensive mice with their normotensive mouse counterparts. Differential TA proteome expression analyses demonstrated that 60 and 91 proteins were significantly up-regulated and down-regulated, respectively (Student’s *t*-test, *P* < 0.05) in response to 28 days of Ang II infusion compared with saline infusion (*Figure 1B*, Supplementary material online, *Figure S3A* and *Table S13*). Functional enrichment analysis (GO biological and cellular processes) based on these significantly changed proteins revealed that aortic structural modulations (GO:0000904, cell morphogenesis involved in differentiation, *P* < 0.003, *Figure 1C*) and calcium regulation (GO:0007010, cytoskeleton organization, *P* < 0.0002, *Figure 1C*) are highly enriched in Ang II-infused mice, whereas mitochondrial-related GO terms (GO:0007005, mitochondrion organization, *P* < 0.0001, *Figure 1C*) are dysregulated in Ang II-infused mice (see Supplementary material online, *Table S14*). Structural proteins (Flnc, Col18a1, and Tagln, *P* < 0.05, *Figure 1B*, Supplementary material online, *Figure S2A*), calcium regulatory proteins (Fbln2, Anxa3, and Lrp1, *P* < 0.05, *Figure 1B*, Supplementary material online, *Figure S2B*), and inflammatory proteins (Cast and Cr1l, *P* < 0.05, *Figure 1B*) were up-regulated, and mitochondrial proteins (Ndufa7, Suclg1, and Ucp1, *P* < 0.05, *Figure 1B*, Supplementary material online, *Figure S2C*) were dysregulated in TA of hypertensive mice in comparison to their normotensive control (see Supplementary material online, *Tables S13* and *S14*). In addition to the TA proteome, Ang II-stimulated HASMC cells exhibited a greater expression of structural proteins (CAPZA1 and LOXL3, *P* < 0.05, *Figure 1D*, Supplementary material online, *Figure S4A* and *Tables S20* and *S21*), inflammatory proteins (BCAP31 and PTGS1, *P* < 0.05, *Figure 1D*, Supplementary material online, *Figure S4A* and *Tables S20* and *S21*), and a lower expression of mitochondrial proteins (ACOT1 and MBLAC2, *P* < 0.05, *Figure 1D*, Supplementary material online, *Figure S4A* and *Tables S20* and *S21*).

We then compared the mouse TA and HASMC proteome with the human aortic remodelling proteome data set from a published human study (from Herrington *et al*.^18^). This human proteome data set identified 1493^18^ proteins, (*Figure 1E*, Supplementary material online, *Tables S2* and *S3*). From this, specific co-identified proteins for each comparison were correlated with hypertension (971 proteins in mouse TA and 1268 proteins in HASMCs), with significantly different expression (*P* < 0.05). Of these commonly identified proteins, the expression of 72 and 26 proteins in mouse TA and HASMCs, respectively, were universally markedly altered by hypertension across the human data set. These altered proteins included up-regulated expression of structural (e.g. Tagln, Flnc, and Capza1), inflammatory proteins (e.g. Bcap31), and calcium regulatory proteins (including Anxa3), while mitochondrial proteins (e.g. Ndufa7, Suclg1, and Acot1) were down-regulated, with other co-identified proteins similar in expression (*Figure 1E* and *F*, Supplementary material online, *Tables S2* and *S3*).

Chronic Ang II infusion induced a similar alteration in the proteome landscape in LV and TA data. In the LV, 110 up-regulated proteins in Ang II-infused mice were identified as associated with networks linked with cell structure and organization, calcium regulation, or inflammatory response proteins, while 38 mitochondrial associated proteins were dysregulated (*P* < 0.05, *Figure 1G*, Supplementary material online, *Figure S3B* and *Table S15*). Functional enrichment analysis (GO biological and cellular processes) based on these differential proteome subsets revealed that cardiac structural modulation (GO:0061061, muscle structure development, *P* < 0.0001, *Figure 1H*) and calcium regulation (GO:1901019, regulation of calcium ion transmembrane transporter activity, *P* < 0.006, *Figure 1H*) are highly enriched in LV from Ang II-infused mice, whereas mitochondrial-related GO terms (GO:0044281, small molecule metabolic process, *P* < 0.0001, *Figure 1H*) are dysregulated in the Ang II-infused LV proteome (see Supplementary material online, *Table S17*). Structural proteins (Myh7, Xirp2, and Flnc, *P* < 0.05, *Figure 1G*, Supplementary material online, *Figure S2D*), calcium regulatory proteins (Sorbs2, Anxa6, and Cdh2, *P* < 0.05, *Figure 1G*, Supplementary material online, *Figure S2E*), and inflammatory response proteins (Cast, Tgm2, and Lta4h, *P* < 0.05, *Figure 1G*) were up-regulated, while mitochondrial proteins (Fth1, Coq8a, Sucla2, and Hspb7, *P* < 0.05, *Figure 1G*, Supplementary material online, *Figure S2F*) were dysregulated in the LV of hypertensive mice compared to normotensive control (see Supplementary material online, *Tables S15* and *S16*). Structural proteins (ADAM17 and TPM1, *P* < 0.05, *Figure 1I*, Supplementary material online, *Figure S4B* and *Tables S22* and *S23*), calcium regulatory proteins (CALU, *P* < 0.05, *Figure 1I*, Supplementary material online, *Figure S4B* and *Tables S22* and *S23*), and inflammatory proteins (IRGQ, *P* < 0.05, *Figure 1I*, Supplementary material online, *Figure S4B* and *Tables S22* and *S23*) were up-regulated, and mitochondrial proteins (ATP5F1D, *P* < 0.05, *Figure 1I*, Supplementary material online, *Figure S4B* and *Tables S22* and *S23*) were dysregulated in HCFs stimulated with Ang II.

We further compared the mouse LV and HCF proteome with hypertrophic human LV proteome data set from Coats *et al*.^19^ co-identifying 24 mouse LV and 9 HCF proteins dysregulated in expression across 127 mouse LV and 153 HCF proteins from Coats’ data set (*P* < 0.05, *Figure 1J* and *K*, Supplementary material online, *Tables S2* and *S3*). These commonly identified and differentially regulated proteins in a hypertrophic human study included cardiac structural associated proteins (including Myh7, Flnc, and Tpm1) and Sorbs2 calcium regulatory proteins (up-regulated in the current study), while mitochondrial proteins (including Sucla2 and Gstm2) decreased in expression compared with our mouse and HCF proteome (*Figure 1J* and *K*, Supplementary material online, *Tables S2* and *S3*).

### 3.2 FPR agonist Cmpd17b reduces blood pressure and overactive sympathetic activity in Ang II-induced hypertensive mice

**Figure 2:**
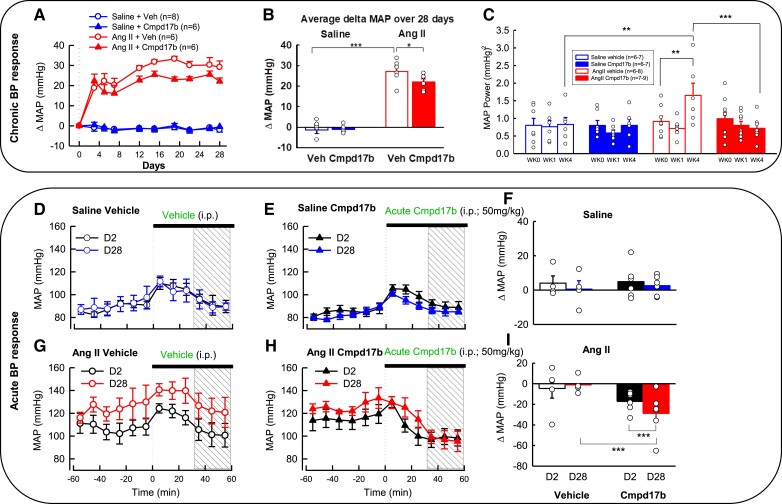
Cmpd17b treatment lowered blood pressure and overactive sympathetic activity in Ang II-induced hypertensive mice. The mean change in MAP (ΔMAP) was recorded by telemetry over 28 days in conscious normotensive mice and hypertensive mice treated with saline vehicle or Cmpd17b (*A*). The average change in MAP (average ΔMAP) over the full 28 days, in normotensive (*n* = 6–8) and hypertensive (*n* = 6) mice treated with vehicle or Cmpd17b (*B*). Spectral analysis was performed to determine mid-frequency MAP power in normotensive (blue, *n* = 6–7) and hypertensive (red, *n* = 6–9) mice treated with vehicle (open bars) or Cmpd17b (filled bars) at baseline, Week 1, and Week 4 (*C*). MAP was recorded before and after (40–60 min) injection of vehicle (*n* = 4, blue unfilled circles) or Cmpd17b (*n* = 6, blue filled triangles) in normotensive mice on Day 2 and Day 28 (*D* and *E*). MAP was recorded before and after (40–60 min) vehicle (*n* = 4, red unfilled circles) and Cmpd17b (*n* = 7, red filled triangles) injection in hypertensive mice on Day 2 and Day 28 (*G* and *H*). The bar graph represents the change in MAP, 30 min (indicated with a square pattern in the line graph) after injection of vehicle or Cmpd17b in normotensive and hypertensive mice at Day 2 vs. Day 28 (*F* and *I*). Dotted vertical lines signify injection of either vehicle or Cmpd17b. Data are presented as mean ± SEM. *P < 0.05, **P < 0.01, and ***P < 0.001 for between-group comparison. Statistical analysis was conducted with a mixed model split-plot ANOVA corrected with Bonferroni and Greenhouse–Geisser adjustments. Ang II, angiotensin II; Veh, vehicle; WK, week; MAP, mean arterial pressure; Cmpd17b, compound 17b; min, minutes; i.p., intraperitoneal; D2, Day 2; D28, Day 28; saline, normotensive mice; Ang II, hypertensive mice.

As shown in *Figure 2A*, there was no mean change in MAP from baseline (ΔMAP) over 28 days with chronic saline infusion in either vehicle- (*n* = 8) or Cmpd17b- (*n* = 6) treated normotensive mice (*P*_Cmpd17b_ > 0.05, *Figure 2A*, Supplementary material online, *Figures S5* and *S6*). After 28 days of Ang II infusion, MAP markedly increased by 31% to 121 mmHg from baseline (92 mmHg) in vehicle-treated mice (*P* < 0.001, *n* = 6, Supplementary material online, *Figures S5* and *S6*). The average change in MAP (average ΔMAP) over the full 28 days was 28 mmHg greater in vehicle-treated hypertensive mice compared with vehicle-treated normotensive mice (ΔMAP: +27 ± 3 vs. −1 ± 2 mmHg, *P*_Ang II_ < 0.001, *Figure 2A* and *B*). The hypertensive response to Ang II infusion was lower in mice treated with Cmpd17b when compared with vehicle-treated hypertensive mice (ΔMAP: +22 ± 4, *P*_Cmpd17b_ = 0.017, *n* = 6, *Figure 2B*, Supplementary material online, *Figures S5* and *S6*).

MAP mid-frequency power is an indicator of sympathetic activity.^23^ At Week 0 (*P*_group_ > 0.05) and Week 1 (*P*_group_ > 0.05), MAP mid-frequency power was comparable in all groups of mice (*Figure 2C*). At Week 4, MAP power was increased from baseline in vehicle-treated hypertensive mice (+1.7 ± 0.3 mmHg^2^ vs. 0.9 ± 0.1 mmHg^2^, *P* = 0.002, *Figure 2C*), whereas there were no detectable differences in the other groups at Week 4 relative to baseline (*P* > 0.05, *Figure 2C*). At Week 4, compared to vehicle-treated normotensive mice (0.8 ± 0.2 mmHg^2^), MAP power was two-fold greater in vehicle-treated hypertensive mice (*P*_Ang II_ = 0.001, *n* = 6, *Figure 2C*). Cmpd17b-treated hypertensive mice displayed a MAP power of 0.7 ± 0.1 mmHg^2^, which was 56% lower than that of vehicle-treated hypertensive mice (*P*_Cmpd17b_ < 0.001, *n* = 6–7, *Figure 2C*).

There was no acute change in blood pressure in response to the vehicle in either normotensive or hypertensive mice treated with the vehicle on Day 2 and Day 28 (*P* > 0.05, *Figure 2D* and *G*). Acute administration of Cmpd17b resulted in a marked depressor response 40–60 min post-injection in hypertensive mice (*P* < 0.006) on both Day 2 and Day 28, but no change in MAP was detected in normotensive mice (*P* > 0.05, 2E, 2H). The acute depressor response to Cmpd17b was potentiated at Day 28 compared to Day 2 in hypertensive mice (ΔMAP: −17 ± 1 mmHg vs. −29 ± 4 mmHg, *P* < 0.001, *n* = 6, *Figure 2I*), but there was no change in MAP in normotensive mice at either time (*P*_Cmpd17b_ > 0.05, *n* = 6, *Figure 2F*). The acute depressor response to Cmpd17b was independent of the angiotensin type 1 (AT1) receptor activation as determined in the acute Ang II and AT1 antagonists, losartan challenges (see Supplementary material online, *Figure S7*).

### 3.3 Impact of dark vs. light periods on hypertensive response

**Figure 3:**
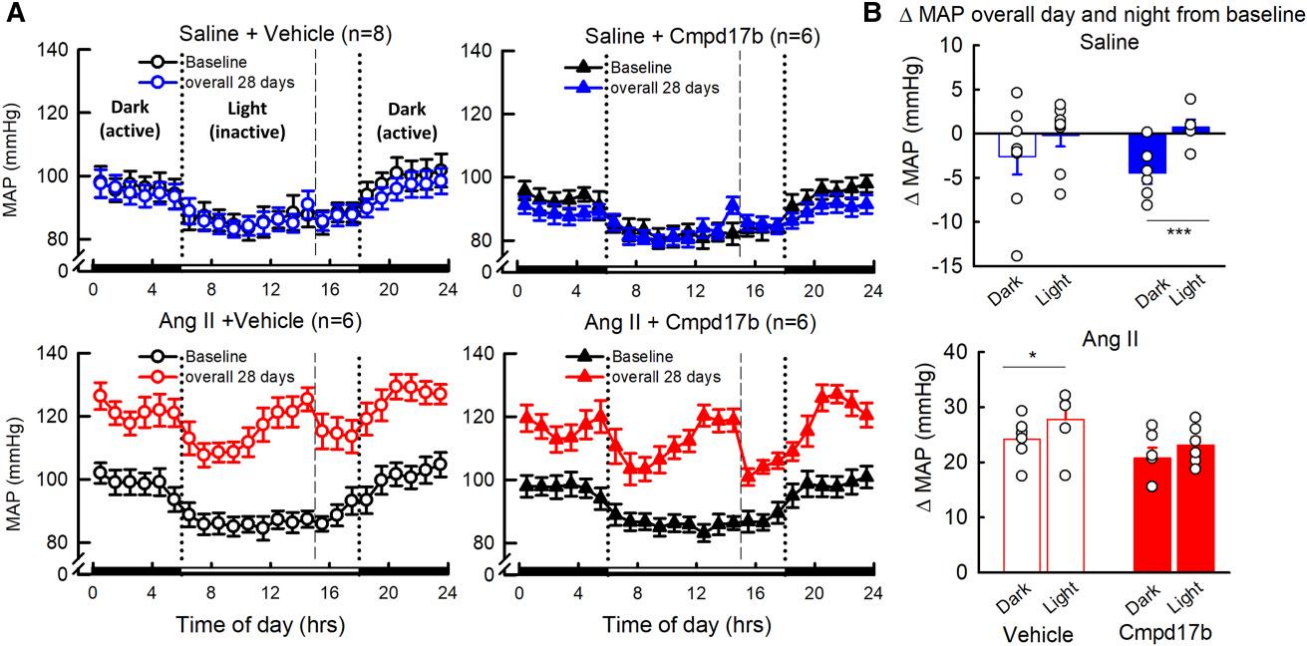
Effect of Cmpd17b or vehicle on MAP in day and night in Ang II-induced hypertensive and normotensive mice. Line graphs show the average hourly MAP over 24 h for vehicle-treated normotensive mice (top left, *n* = 8, blue unfilled circles), Cmpd17b-treated normotensive mice (top centre, *n* = 6, blue filled triangles), vehicle-treated hypertensive mice (bottom left, *n* = 6, red unfilled circles), and Cmpd17b-treated hypertensive mice (bottom centre, *n* = 6, red filled triangles) at baseline (black) and over 28 days (*A*). Dotted vertical lines signify lights on–off. Dashed vertical lines signify injection of either vehicle or Cmpd17b. The dark (active) period is indicated by a black bar on a time axis, and the light (inactive) period is indicated by a white bar on the time axis. Histograms indicate the average change in MAP from baseline during the dark and light period over the 28 days of chronic treatment with saline, top right, or Ang II, bottom right (*B*). Data presented as mean ± SEM. *P < 0.05 and ***P < 0.001 for between-group comparison. Statistical analysis was conducted with between-group split-plot ANOVA of average hourly values corrected with Bonferroni and Greenhouse–Geisser adjustments. MAP, mean arterial pressure; Ang II, angiotensin II; Cmpd17b, compound 17b; hrs, hours; saline, normotensive mice; Ang II, hypertensive mice.

MAP averaged over 28 days was higher during the dark (active) period than during the light (inactive) period for all groups (*Figure 3A* and *B*, *P*_group_ < 0.001). The change in MAP from baseline was comparable during the dark and light periods over the entire 28 days in Cmpd17b-treated hypertensive mice (*P*_Cmpd17b_ > 0.05). The change in MAP from baseline over the 28 days was lower during the dark compared to the light period for vehicle-treated hypertensive mice and Cmpd17b-treated normotensive mice (*P* < 0.05, *n* = 6–8, *Figure 3A* and *B*).

### 3.4 FPR agonist Cmpd17b blunts Ang II-induced adverse cardiac dysfunction and cardiorenal remodelling

**Figure 4:**
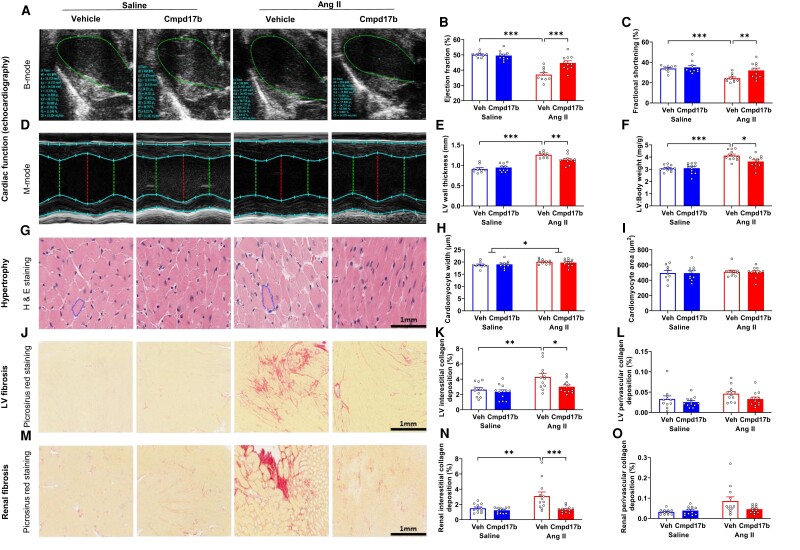
Chronic Cmpd17b treatment prevented adverse cardiac dysfunction and cardiorenal remodelling in Ang II-induced hypertensive mice. Two-dimensional, long-axis echocardiography of the LV was performed to measure EF, and M-mode echocardiography was performed to measure LV FS and wall thickness. The representative echocardiography images show LV long-axis and M-mode (*A–E*). LV weight normalized to body weight (mg/g) was recorded (*F*). H&E staining was used to quantify the cardiomyocyte’s width and area. Representative images of LV showed cardiomyocytes (*G–I*). PSR stain quantified interstitial and perivascular fibrotic area of LV and shown as red-stained collagen in representative images (*J–L*). PSR staining was used to quantify interstitial and perivascular fibrotic area of the kidney and shown as red-stained collagen in representative images (*M–O*). Histograms (*B–O*) represent vehicle-treated normotensive mice (*n* = 9–11, blue unfilled), Cmpd17b-treated normotensive mice (*n* = 10–12, blue filled), vehicle-treated hypertensive mice (*n* = 10–12, red unfilled), and Cmpd17b-treated hypertensive mice (*n* = 11–12, red filled). The scale bar in black represents 1 mm. Results presented as mean ± SEM. Statistical analysis was performed using a two-way ANOVA followed by Bonferroni significant difference *post hoc* test. *P < 0.05, **P < 0.01, and ***P < 0.001 for differences between groups. Ang II, angiotensin II; Veh, vehicle; H&E, haematoxylin and eosin; Cmpd17b, compound 17b; LV, left ventricle; PSR, picrosirius red; saline, normotensive mice; Ang II, hypertensive mice.

Ejection fraction (EF, 26%, *P*_Ang II_ < 0.001, *n* = 9–11, *Figure 4A* and *B*, Supplementary material online, *Table S4*) and fractional shortening (FS, 28%, *P*_Ang II_ < 0.001, *n* = 9–10, *Figure 4A* and *C*, Supplementary material online, *Table S5*) were lower in vehicle-treated hypertensive mice compared with vehicle-treated normotensive mice analysed from echocardiography. Cmpd17b improved FS (+31%, *P*_Cmpd17b_ > 0.05, *n* = 10, *Figure 4A* and *C*, Supplementary material online, *Table S5*) and EF (+20%, *P*_Cmpd17b_ < 0.001, *n* = 11, *Figure 4A* and *B*, Supplementary material online, *Table S4*) in vehicle-treated hypertensive mice. LV wall thickness was greater in vehicle-treated hypertensive mice compared with that in vehicle-treated normotensive mice (+39%, *P*_Ang II_ < 0.001, *n* = 11–12, *Figure 4D* and *E*, Supplementary material online, *Table S5*). Cmpd17b treatment reduced wall thickness (−10%, *P*_Cmpd17b_ = 0.007, *n* = 10, *Figure 4D* and *E*, Supplementary material online, *Table S5*) in vehicle-treated hypertensive mice. LV weight normalized to body weight (LV/BW) was 34% greater in vehicle-treated hypertensive mice compared with that in vehicle-treated normotensive mice (4.1 ± 0.1 mg/g vs. 3.1 ± 0.1 mg/g, *P*_Ang II_ < 0.001, *n* = 11–12, *Figure 4F*), which was 12% lower in Cmpd17b-treated hypertensive mice (3.6 ± 0.1 mg/g, *P*_Cmpd17b_ = 0.026, *n* = 12, *Figure 4F*, Supplementary material online, *Table S6*). Cardiomyocyte width was greater in Ang II-infused mice compared with that in normotensive mice (*P*_Ang II_ = 0.017, *Figure 4G* and *H*), which was comparable with Cmpd17b (*P*_Cmpd17b II_ > 0.05, *Figure 4G* and *H*). No differences were detected in normotensive mice and Ang II-infused mice for the cardiomyocyte area (*P*_Ang II_ > 0.05, *Figure 4G* and *I*). LV interstitial and perivascular collagen depositions were comparable in normotensive mice treated with vehicle and Cmpd17b (*P* > 0.05, *n* = 11–12, *Figure 4J–L*). Interstitial collagen deposition was 63% greater in vehicle-treated hypertensive mice compared to that in vehicle-treated normotensive mice (*P*_Ang II_ = 0.006, *Figure 4K*). Interstitial collagen deposition was 30% lower in Cmpd17b-treated hypertensive mice compared to that in vehicle-treated hypertensive mice (3.0 ± 0.2% vs. 4.3 ± 0.5%, *n* = 12, *P*_Cmpd17b_ = 0.049, *Figure 4K*). LV perivascular collagen deposition was not different between the groups (*P*_Ang II_ > 0.05, *P*_Cmpd17b_ > 0.05, *Figure 4L*). There was no significant difference in renal interstitial collagen deposition (*P* = 0.544, *n* = 11–12, *Figure 4M* and *N*) and perivascular collagen deposition (*P* > 0.05, *n* = 11–12, *Figure 4M* and *O*) between normotensive mice treated with vehicle and Cmpd17b. Vehicle-treated hypertensive mice exhibited approximately two-fold greater interstitial collagen deposition (+3.1 ± 0.5%, *P*_Ang II_ = 0.003, *Figure 4M* and *N*) and perivascular collagen deposition (+0.09 ± 0.02%, *P*_Ang II_ = 0.011, *n* = 12, *Figure 4M* and *O*) in kidney compared to vehicle-treated normotensive mice. Cmpd17b showed 56% lower renal interstitial collagen deposition (1.3 ± 0.1%, *P*_Cmpd17b_ < 0.001, *n* = 12, *Figure 4M* and *N*) and 50% lower perivascular collagen deposition (0.04 ± 0.01%, *P*_Cmpd17b_ = 0.049, *Figure 4M* and *O*) than in vehicle-treated hypertensive mice. Kidney weight normalized to body weight was 13% lower in vehicle-treated hypertensive mice compared to that in vehicle-treated normotensive mice (5.4 ± 0.2 mg/g vs. 6.2 ± 0.1 mg/g, *P*_Ang II_ = 0.007). In addition, kidney weight was greater in Cmpd17b-treated hypertensive mice compared to that in vehicle-treated hypertensive mice (*P*_Cmpd17b_ = 0.048, Supplementary material online, *Figure S8*). Glomerulosclerosis index (GSI) score was not different among all groups (*P* < 0.05, Supplementary material online, *Figure S8*).

### 3.5 FPR agonist Cmpd17b blunts Ang II-induced adverse vascular remodelling and function

**Figure 5:**
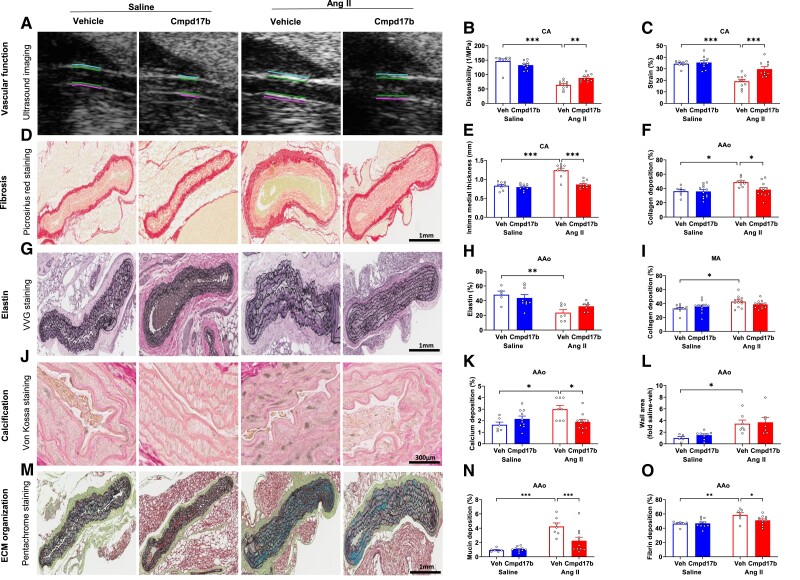
Chronic Cmpd17b treatment prevents adverse vascular remodelling in Ang II-induced hypertensive mice. Ultrasound imaging of the carotid artery was performed to measure distensibility, strain, and wall thickness. The representative images showed intima medial thickness (*A*–*C* and *E*). Quantification of the collagen (fibrotic) area of the abdominal aorta was performed by PSR stain. Red-stained collagen is shown in the representative images of the vessel (*D* and *F*). Quantification of elastin was performed by VVG stain. Representative images of vessel elastin content show black-stained elastin lining (*G* and *H*). The collagen (fibrotic) area of the mesenteric artery was quantified using a PSR stain (*I*). Calcification was quantified as the percentage area of calcium deposition in the abdominal aorta using the Von Kossa stain (*J* and *K*). The wall area of the abdominal aorta was quantified (*L*). Pentachrome staining was used to quantify mucin and fibrin area of abdominal aorta and shown as blue-stained mucin and red-stained fibrin in representative images (*M–O*). Histograms (*B–O*) represent vehicle-treated normotensive mice (*n* = 5–11, blue unfilled), Cmpd17b-treated normotensive mice (*n* = 9–12, blue filled), vehicle-treated hypertensive mice (*n* = 7–12, red unfilled), and Ang II-infused Cmpd17b-treated mice (*n* = 7–12, red filled). The scale bar in black represents 1 mm for pentachrome, PSR, and VVG stain images and 300 µm for Von Kossa stain images. Results presented as mean ± SEM. Statistical analysis was performed using a two-way ANOVA followed by Bonferroni significant difference *post hoc* test. *P < 0.05, **P < 0.01, and ***P < 0.001 for differences between groups. Ang II, angiotensin II; Veh, vehicle; VVG, Verhoeff-van Gieson; Cmpd17b, compound 17b; PSR, picrosirius red; saline, normotensive mice; Ang II, hypertensive mice; ECM, extracellular matrix; CA, carotid artery; AAo, abdominal aorta; MA, mesenteric artery.

Carotid distensibility (56%, *P*_Ang II_ < 0.001, *n* = 9–10, *Figure 5A* and *B*) and strain (44%, *P*_Ang II_ < 0.001, *n* = 9–10, *Figure 5A* and *C*, Supplementary material online, *Table S7*) were lower, and intima medial wall thickness (49%, *P*_Ang II_ < 0.001, *n* = 9–10, *Figure 5A* and *E*, Supplementary material online, *Table S7*) was greater in vehicle-treated hypertensive mice compared with those in vehicle-treated normotensive mice analysed from ultrasound imaging. Cmpd17b improved distensibility (+38%, *P*_Cmpd17b_ = 0.002, *n* = 10, *Figure 5A* and *B*), strain (+54%, *P*_Cmpd17b_ < 0.001, *n* = 10, *Figure 5A* and *C*), and wall thickness (−30%, *P*_Cmpd17b_ < 0.001, *n* = 10, *Figure 5A* and *E*) in hypertensive mice (see Supplementary material online, *Table S7*). Collagen deposition in the abdominal aorta was not different between vehicle-treated normotensive mice and Cmpd17b-treated normotensive mice (36 ± 2% vs. 36 ± 3%, *P* > 0.05, *n* = 8–12, *Figure 5D* and *F*, Supplementary material online, *Figure S9*). Collagen deposition was 35% greater in vehicle-treated hypertensive mice compared to that in vehicle-treated normotensive mice (49 ± 2%, *P*_Ang II_ = 0.019, *n* = 9, *Figure 5D* and *F*, Supplementary material online, *Figure S9*). Collagen deposition was lower in Cmpd17b-treated hypertensive mice compared with that in vehicle-treated hypertensive mice (38 ± 3%, *P*_Cmpd17b_ = 0.039, *n* = 12, *Figure 5D* and *F*, Supplementary material online, *Figure S9*). Collagen deposition in the mesenteric artery was 30% greater in vehicle-treated hypertensive mice compared with normotensive counterparts (43 ± 2% vs. 33 ± 2, *P*_Ang II_ = 0.002, *n* = 11–12, *Figure 5I*). Collagen deposition (39 ± 2%) was comparable between Cmpd17b-treated hypertensive mice and vehicle-treated hypertensive mice (*P*_Cmpd17b_ > 0.05, *n* = 12, *Figure 5I*). Elastin percentage was not different between vehicle-treated normotensive mice and Cmpd17b-treated normotensive mice (*P*_Cmpd17b_ > 0.05, *n* = 5–9, *Figure 5G* and *H*, Supplementary material online, *Figure S9*). Elastin percentage was 51% lower in vehicle-treated hypertensive mice compared with that in vehicle-treated normotensive mice (24 ± 4% vs. + 48 ± 5%, *P*_Ang II_ = 0.003, *n* = 5–7, *Figure 5G* and *H*, Supplementary material online, *Figure S9*). Elastin percentage (32 ± 2%) was comparable between Cmpd17b-treated hypertensive mice and vehicle-treated hypertensive mice (*P*_Cmpd17b_ > 0.05, *n* = 7, *Figure 5G* and *H*, Supplementary material online, *Figure S9*). Calcium deposition in the abdominal aorta was comparable between vehicle-treated normotensive mice and normotensive mice + Cmpd17b (*P* > 0.05, *n* = 6–10, *Figure 5J* and *K*, Supplementary material online, *Figure S9*). Calcium deposition was +80% greater in vehicle-treated hypertensive mice relative to that in vehicle-treated normotensive mice (+3.0 ± 0.3% vs. 1.7 ± 0.2%, *P*_Ang II_ = 0.016, *n* = 6–8, *Figure 5J* and *K*, Supplementary material online, *Figure S9*). Cmpd17b caused an ∼38% lower calcium deposition (−1.9 ± 0.2%, *P*_Cmpd17b_ = 0.025, *n* = 8–11, *Figure 5J* and *K*, Supplementary material online, *Figure S9*) than in vehicle-treated hypertensive mice. The wall area of the abdominal aorta did not change in vehicle vs. Cmpd17b-treated normotensive mice (*P*_Cmpd17b_ > 0.05, *n* = 9, *Figure 5L*). The wall area was 3.5-fold higher in vehicle-treated hypertensive mice (*P*_Ang II_ = 0.045, *n* =7, *Figure 5L*) compared to that in normotensive mice, which were not changed by Cmpd17b (*P*_Cmpd17b_ > 0.05, *n* = 7, *Figure 5L*). Mucin and fibrin deposition in the abdominal aorta was not different between vehicle-treated normotensive mice and Cmpd17b-treated normotensive mice (*P*_Cmpd17b_ > 0.05, *n* = 6–10, *Figure 5M–O*). Mucin and fibrin deposition was four-fold and three-fold greater in vehicle-treated hypertensive mice compared with that in vehicle-treated normotensive mice, respectively (*P*_Ang II_ < 0.05, *n* = 6–7, *Figure 5M–O*). Mucin percentage was 47% lower in Cmpd17b-treated hypertensive mice compared with that in vehicle-treated hypertensive mice (*P*_Cmpd17b_ = 0.001, *n* = 7–11, *Figure 5M* and *N*). Cmpd17b caused an ∼13% lower fibrin deposition (*P*_Cmpd17b_ = 0.022, *n* = 7–11, *Figure 5M* and *O*) than in vehicle-treated hypertensive mice.

### 3.6 FPR agonist Cmpd17b attenuated Ang II-induced changes in the cardiac and vascular proteome

**Figure 6:**
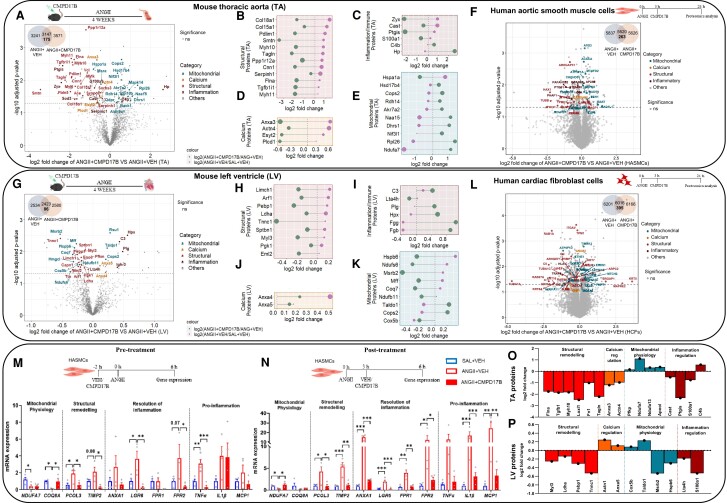
Cmpd17b attenuated cardiac and vascular proteome in Ang II-induced hypertensive mice. Volcano plots (with corresponding Venn diagram in the inset) and lollipop chart comparing structural (red), inflammatory (brown), calcium regulatory (orange), and mitochondrial (blue) proteins in TA (*A–E*) and LV (*G–K*) of hypertensive mice treated with vehicle and Cmpd17b. Lollipop chart of TA proteome (*B–E*) and LV proteome (*H–K*) displayed up-regulated proteins as pink dots and down-regulated proteins as green dots. Ang II-stimulated HASMCs (*F*) and HCFs (*L*) treated with Cmpd17b or vehicle before subjected to proteomic analysis. Volcano plots (with corresponding Venn diagram in the inset) display proteins comparing structural (red), inflammatory (brown), calcium regulatory (orange), and mitochondrial (blue) proteins in human cell lines (*M* and *N*). Ang II-stimulated HASMCs treated with Cmpd17b (pre-treatment, *M*, and post-treatment, *N*) were subjected to gene expression analysis, including mitochondrial physiology, structural remodelling, resolution of inflammation, and pro-inflammation. Histograms (*M* and *N*) represent HASMCs treated with vehicle (*n* = 4, blue unfilled), Ang II-stimulated HASMCs treated with vehicle (*n* = 3–4, red unfilled), and Ang II-stimulated HASMCs treated with Cmpd17b (*n* = 3–4, red filled). The differential protein expression in TA (*O*) and LV (*P*) of Cmpd17b-treated hypertensive mice exhibits inverse impact on human data sets. For gene expression, statistical analysis was performed using a one-way ANOVA followed by Bonferroni significant difference *post hoc* test. *P < 0.05, **P < 0.01, and ***P < 0.001 for differences between groups. Veh, vehicle; Ang II, angiotensin II; AngII + Veh, vehicle-treated hypertensive mice; AngII + Cmpd17b, Cmpd17b-treated hypertensive mice; Cmpd17b, compound 17b; HCFs, human cardiac fibroblasts; HASMCs, human aortic smooth muscle cells.

Given the robust impact of Cmpd17b on cardiac and vascular function and remodelling, its corresponding impact on the proteome was sought. A total of 3571 and 2580 proteins were identified in TA (see Supplementary material online, *Table S11*) and LV (see Supplementary material online, *Table S12*) samples, respectively. Of these, 3147 TA proteins and 2423 LV proteins were identified in both vehicle-treated hypertensive mice and Cmpd17b-treated hypertensive mice, respectively, out of which 175 TA proteins and 86 LV proteins were dysregulated in expression across both vehicle-treated hypertensive and Cmpd17b-treated hypertensive mice (*P* < 0.05, *Figure 6A* and *G*, Supplementary material online, *Figure S3A* and *B* and *Tables S13* and *S15*). Proteomic analysis of HASMCs and HCFs stimulated with Ang II co-identified ∼6000 proteins in Cmpd17b and vehicle treatment (see Supplementary material online, *Figure S4* and *Tables S20–S23*). Out of these commonly identified proteins, 263 HASMC and 395 HCF proteins were different in Ang II-stimulated cells treated with Cmpd17b vs. vehicle (*P* < 0.05, *Figure 6F* and *L*, Supplementary material online, *Figure S4* and *Tables S20–S23*). Based on differential expression in vehicle-treated hypertensive mice, networks associated with structural organization, calcium regulation, and inflammatory response proteins of mouse LV and TA were down-regulated in Cmpd17b-treated hypertensive mice (60 vs. 52 in TA, *Figures 1B* and *6A*, and 110 vs. 58 in LV, *Figures 1G* and *6G*, Supplementary material online, *Tables S12* and *S15*). For TA region, structural (Col18a1, Myh11, and Tagln, *Figure 6B*), inflammatory (Cast, C4b, and Ptgis, *Figure 6C*), and calcium regulatory proteins (Anxa3, Plcd, and Esyt2, *Figure 6D*) were shifted in their expression in response to Cmpd17b hypertensive treatment compared with vehicle-treated hypertensive mice (see Supplementary material online, *Tables S13* and *S17*). From a global proteome perspective, the alteration in protein profiles observed in Cmpd17b-treated hypertensive TA tissue indicates a distinction in the hypertensive tissue proteome compared to vehicle treatment (see Supplementary material online, *Figure S3A*). Cmpd17b-mediated alterations in the hypertensive global tissue proteome supports the vasoprotective mechanism of action of Cmpd17b via targeting remodelling-specific protein networks towards normotensive patterns associated with physiological structural, metabolic, and inflammatory processes. Further, for LV tissue, structural proteins (Ldha, Tnnc1, and Pebp1, *Figure 6H*) were reduced in expression while inflammatory associated proteins (C3, Lta4h, and Plg, *Figure 6I*) were reduced in LV of Cmpd17b-treated hypertensive mice compared with vehicle-treated hypertensive mice. Calcium regulatory proteins (Anxa4, Anxa5) in LV were higher in both Cmpd17b-treated hypertensive mice and vehicle-treated hypertensive mice (*P* = 0.04, *Figure 6J*, Supplementary material online, *Tables S15* and *S18*). We further demonstrate alterations in global protein profiles of Cmpd17b-treated hypertensive LV tissue proteome (see Supplementary material online, *Figure S3B*).

Dysregulated mitochondrial proteins in hypertensive mice treated with vehicle revealed these altered proteomes were not evident in hypertensive mice treated with Cmpd17b (91 vs.74 in TA, *Figures 1B* and *6A*, and 38 vs. 28 in LV, *Figures 1G* and *6G*, Supplementary material online, *Tables S13* and *S15*). Aortic mitochondrial proteins (Naa15, Rpl26, and Ndufa7, *Figure 6E*) were higher in Cmpd17b-treated hypertensive mice compared with vehicle-treated hypertensive mice (see Supplementary material online, *Tables S13* and *S17*). Dysregulated cardiac mitochondrial proteins (Taldo, Msrb2, and Ndufb11, *Figure 6K*) in vehicle-treated hypertensive mice were not evident in Cmpd17b-treated hypertensive mice (see Supplementary material online, *Tables S15* and *S18*).

In Ang II-stimulated HASMCs, Cmpd17b restored the expression of protein associated with structural (MYH11, CDC42), inflammatory (BCAP31, PTGS1), calcium regulatory (CAMSAP2), and mitochondrial (IBA57, ATP5F1A) processes towards physiological levels (*P* < 0.05, *Figure 6F*, Supplementary material online, *Table S20*). Further, the Ang II-stimulated HCFs displayed changes in expression of structural (TUBA1A, MYL12A), inflammatory (TRAF2, BCL7C), and calcium regulatory (CHP1, ANXA3) proteins that were also restored with Cmpd17b treatment (*P* < 0.05, *Figure 6L*, Supplementary material online, *Table S22*). Cmpd17b prevented changes in expression of mitochondrial proteins (IBA57, ATP5F1A) in HASMCs and (COX6C, ATP5F1E) in HCFs stimulated with Ang II (*P* < 0.05, *Figure 6F* and *L*, Supplementary material online, *Tables S20* and *S22*). Expression of genes involved in mitochondrial function (*NDUFA7*, *COQ8A*), structural remodelling (*PCOL3*), resolution of inflammation (*LGR6*, *FPR2*), and pro-inflammation (*TNFα*) was dysregulated in Ang II-stimulated HASMCs pre-treated with vehicle (*P* < 0.05, *n* = 3–4, *Figure 6M*, Supplementary material online, *Table S10*). Post-treatment with vehicle altered expression of genes involved in mitochondrial function (*NDUFA7*, *COQ8A*), structural remodelling (*PCOL3*, *TIMP2*), resolution of inflammation (*ANXA1*, *LGR6*, *FPR1*, and *FPR2*), and pro-inflammation (*IL1β*, *MCP1*) was dysregulated in Ang II-stimulated HASMCs (*P* < 0.05, *n* = 3–4, *Figure 6N*, Supplementary material online, *Table S10*). Cmpd17b reduces expression of genes involved in mitochondrial function (*NDUFA7*), structural remodelling (*PCOL3*, *TIMP2*), resolution of inflammation (*ANXA1, LGR6*, *FPR1*, and *FPR2*), and pro-inflammation (*IL1β*, *MCP1*) in HASMCs stimulated with Ang II.

We then examined whether those mouse TA and LV proteins in which Cmpd17b blunted the hypertension-induced changes were altered in human hypertension^18,19^ We identified 12 expressed proteins altered in human myocardial data set from Coats *et al*.^19^ compared with 86 expressed LV proteins identified as altered by Cmpd17b in murine hypertension (*P* < 0.05, Supplementary material online, *Tables S8* and *S3*). However, of the 175 expressed TA proteins identified as altered by Cmpd17b in murine hypertension, 117 of these were common in human aortic remodelling data set from Herrington *et al*.^18^ (*P* < 0.05, Supplementary material online, *Tables S8* and *S3*). These include proteins co-identified in human study dysregulated in TA (*P* < 0.05, Pgm5, Flna, Rab7a, Anxa6, Lrp1, Bcam, Rras, Aoc3, and Npepps) and in LV (*P* < 0.05, Anxa5, Arf1, Prdx6, Pgk1, and Actn1) of Ang II-infused mice following Cmpd17b (see Supplementary material online, *Tables S8* and *S3*). Functional enrichment analyses (*P* < 0.001) of these proteins identified in both human studies and the current study revealed that vasculature function/development, extracellular matrix (ECM) organization, and peptide metabolic processes (TA proteome) while focal adhesion, cardiac muscle contraction, oxidative phosphorylation, and associated metabolic processes (LV proteome) characterized the proteome affected in Cmpd17b-treated hypertensive mice (see Supplementary material online, *Figure S10*).

In the murine proteome, Cmpd17b reduced TA expression of structural proteins (Flna, Myh10) and calcium regulatory proteins (Actn4) and exhibited higher expression of mitochondrial proteins (Ndufa7, Ndufa13) in hypertensive mice (*Figure 6O*). In addition, Cmpd17b resulted in reduced LV expression of proteins associated with structural and contractile components of the heart (Myl3, Ldha) and further exhibited higher expression of mitochondrial proteins (Taldo1) in hypertensive mice (*Figure 6P*). This differential expression induced by Cmpd17b in our hypertensive murine data set was the inverse of the impact of hypertension in the human data sets. For example, proteins important for vascular structure (FLNA) and calcium regulation (ACTN4) were up-regulated, and mitochondrial function (COX6B1) was down-regulated, in human hypertension. Similarly, proteins important for LV structure (MYL3), and mitochondrial function (COX5B), were up-regulated and down-regulated, respectively, in human hypertension. This suggests Cmpd17b treatment influences distinct proteome expression to support remodelling of the cellular component process, and of molecular function, in hypertension (see Supplementary material online, *Figure S11*).

### 3.7 FPR agonist Cmpd17b attenuates cardiac mitochondrial dysregulation in Ang II-induced hypertensive mice.

**Figure 7:**
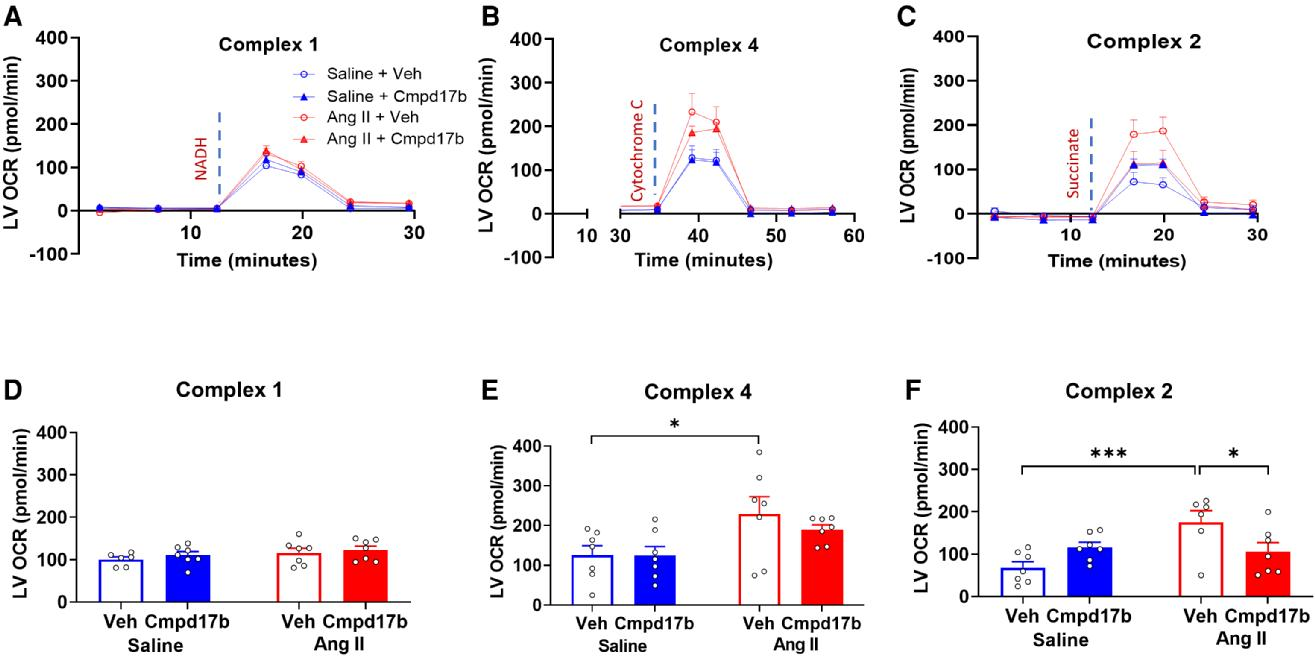
Cmpd17b attenuates cardiac mitochondrial dysregulation in Ang II-induced hypertensive mice. OCR of complexes I, II, and IV was measured for 60 min in mitochondria isolated from LV of normotensive and hypertensive mice treated with vehicle and Cmpd17b (*A–C*). Histogram (*D–F*) represents OCR of complexes I, II, and IV of vehicle-treated normotensive mice (*n* = 6–7, blue unfilled), Cmpd17b-treated normotensive mice (*n* = 7, blue filled), vehicle-treated hypertensive mice (*n* = 6–7, red unfilled), and Cmpd17b-treated hypertensive mice (*n* = 7, red filled). Results presented as mean ± SEM. Statistical analysis was performed using a two-way ANOVA followed by Bonferroni significant difference *post hoc* test. *P < 0.05 and ***P < 0.001 for differences between groups. OCR, oxygen consumption rate; Ang II, angiotensin II; Veh, vehicle; Cmpd17b, compound 17b; LV, left ventricle.

Cardiac Complex 1 OCR was not different between all groups (*P* > 0.05, *n* = 6–7, *Figure 7A* and *D*). Complex 4 OCR was higher in the vehicle-treated hypertensive mice compared to that in vehicle-treated normotensive mice (229 ± 46 pmol/min vs. 125 ± 23 pmol/min, *P* < 0.05, *n* = 7, *Figure 7B* and *E*). The effect was comparable to OCR in Cmpd17b-treated hypertensive mice (189 ± 12 pmol/min, *P* > 0.05, *n* = 7, *Figure 7B* and *E*). Complex 2 OCR was greater in the vehicle-treated hypertensive mice compared to that in vehicle-treated normotensive mice (175 ± 27 pmol/min vs. 68 ± 14 pmol/min, *P* < 0.05, *n* = 6–7, *Figure 7C* and *F*). Cmpd17b treatment lowered Complex 2 OCR (106 ± 21 pmol/min, *P*_Cmpd17b_ = 0.018, *n* = 7, *Figure 7C* and *F*) in vehicle-treated hypertensive mice.

## 4. Discussion

There is an ongoing search for novel pharmacological interventions to attenuate end-organ damage in hypertension, in addition to the management of blood pressure. This study demonstrated for the first time that a prototype, small-molecule FPR agonist, Cmpd17b, prevents hypertension-induced cardiovascular and renal damage. Cmpd17b moderately lowers blood pressure and markedly improves structural remodelling and mitochondrial physiology in the heart and vasculature. Our mouse proteomic data indicate that Cmpd17b selectively limits hypertension-induced remodelling of both TA and LV regions and has a positive impact on proteins that are implicated in human hypertension.

We have shown that chronic Ang II infusion induced a hypertensive response and associated end-organ damage, selectively remodelling the heart and vascular proteome, and impairment of cardiovascular function, consistent with human hypertension. We demonstrate this through quantitative tissue proteomics in addition to informatics, enrichment, and functional analyses. Such findings highlight the molecular impact of hypertensive cardiac and aortic landscape and the reversal of the changes in protein networks in response to Cmpd17b treatment. This proteomic cartography provides a comprehensive approach to understanding complex, global disease-remodelled cardiac and aortic proteome, and the translational potential of proteomics^24^ from complex tissues to identify molecular insights into hypertension^18,19^ and its treatment, in addition to correlation analyses with human hypertension.

Ang II induces hypertension by acting on AT1 receptors to cause vasoconstriction, sympathetic nerve stimulation, increased aldosterone biosynthesis, and increased sodium retention by the kidney.^25^ Administration of a single dose of Cmpd17b promptly lowered MAP acutely on Day 2, and the MAP lowering effect was potentiated on Day 28. This suggests that there is little or no tolerance to the actions of Cmpd17b over the 28-day treatment period. The acute vasodepressor effect of Cmpd17b *in vivo* is consistent with relaxation previously reported in isolated aorta and mesenteric arteries.^10^ The potentiated vasodilator response to Cmpd17b on Day 28 compared to Day 2 suggests a vascular amplifying effect with chronic Ang II infusion, which may be due to more vascular remodelling and narrower arterial lumen on Day 28 compared to Day 2.^26^ Chronic Cmpd17b administration attenuated the development of hypertension in Ang II-infused mice. Its ability to blunt cardiovascular fibrosis may in turn attenuate Ang II-induced hypertension or *vice versa*. MAP power, an indicator of sympathetic nerve activity, was elevated by Week 4 in vehicle-treated hypertensive mice compared with that in saline-infused mice, but not in Cmpd17b-treated hypertensive mice, suggesting Cmpd17b may prevent the Ang II-induced increase in sympathetic vasomotor tone. These findings suggest that the chronic blood pressure-lowering effect of Cmpd17b may be due to reduced sympathetic overactivity and improved vascular distensibility in hypertensive mice. MAP power is, however, an indirect measure of sympathetic tone and should therefore be interpreted with caution.

Cardiovascular hypertrophy and fibrosis are characteristic structural changes that develop during hypertension.^27^ Previous studies demonstrated that Ang II-dependent hypertension is associated with myocardial enlargement and collagen accumulation in the LV and aorta.^28–30^ In our study, fibrosis (demonstrated by collagen deposition) was elevated in the LV and abdominal aorta of hypertensive mice. Our cardiac and aortic structural subproteome analysis demonstrated that fibrotic proteins were up-regulated in Ang II-induced hypertensive mice, consistent with the observed pathology. This is also consistent with an elevation in the expression of fibrotic genes and proteins in the LV^31^ and abdominal aorta^32^ of Ang II-infused mice. Cmpd17b reduced collagen deposition in the LV and abdominal aorta to a level observed in normotensive mice (i.e. returned to baseline) indicating its selective and targeted anti-fibrotic effect. Such observations are consistent with our previous findings, where Cmpd17b reduced LV collagen deposition in mice after MI.^11^ In addition to fibrosis, up-regulated fibrosis-associated proteins (i.e. Lmo7) were normalized in LV and TA of Cmpd17b-treated hypertensive mice. These observations suggest that Cmpd17b may ameliorate adverse structural remodelling in hypertensive mice accompanied by reduced fibrotic proteins and collagen deposition in LV and TA. Comparison with human data sets suggests Cmpd17b affects proteins that are relevant to human hypertension.

We observed that LV weight was greater in hypertensive mice than their normotensive counterparts, indicating LV hypertrophy results from chronic Ang II infusion, consistent with previous report.^30^ LV cardiomyocyte hypertrophy, specifically cardiomyocyte width (an early sign of enlarged cardiomyocytes), in hypertensive mice was also evident in response to Ang II infusion. Previous studies using Ang II infusion at a higher dose and longer duration (1.4 mg/kg/day, 8 weeks) showed greater cardiomyocyte area in LV of C57BL/6 mice.^33^ In addition, hypertrophic (Myh7, Limch1), contractile (Tnnc1), and inflammatory (Lta4h) proteins were up-regulated in the LV and ECM (Col15a1, Flna); contractile (Tagln) and inflammatory (Cast) proteins were up-regulated in TA of vehicle-treated hypertensive mice, consistent with previous reports.^31^ Interestingly, Cmpd7b normalized hypertrophic, contractile, and inflammatory proteins in hypertensive mice. Importantly, the above-mentioned structural improvements with Cmpd17b may have led to functional improvement.

Our echocardiographic analysis demonstrated that Ang II infusion resulted in a greater LV wall thickness and moderately lower EF coupled with an increased lung weight, indicating cardiac dysfunction^34^ and pulmonary congestion.^35^ These findings are relatively more consistent with the development of heart failure with moderately reduced ejection fraction (HFmrEF) due to hypertension. Cmpd17b prevented this cardiac dysfunction, as reflected by lower wall thickness and improved HFmrEF, but had no marked impact on lung weight. Our HCF proteomic analysis demonstrated that expression of the cytoskeleton protein Msn was greater in Ang II-stimulated HCFs, consistent with its critical role in pulmonary oedema caused by heart failure.^36^ Interestingly, our data demonstrated that Cmpd17b reduces Msn expression in human hypertensive cardiac fibroblasts, suggesting that FPR agonism may lead to a decrease in Msn-mediated re-arrangements of cytoskeleton.

Our study demonstrated that Cmpd17b attenuated LV wall thickness and LV mass, although no differences in cardiomyocyte area were observed. The exact mechanism for this discrepancy between LV hypertrophy and cardiomyocyte hypertrophy is, however, beyond the scope of the present study. We speculate that this might be due to the excessive infiltration of inflammatory cells into hypertensive LV, favouring progression towards fibrosis. Consistent with this, previous studies reported that Ang II infusion (2.8 mg/kg/day) for 7 days resulted in marked cellular infiltration of leucocytes, macrophages, and accumulation of myofibroblasts, and endothelial cells in the heart.^37^ The increased LV mass and thickness could reflect increases in non-cardiomyocyte cell types in the hypertensive heart, such as immune cells and fibroblasts. On the other hand, lower circulating blood leucocytes have been observed in hypertensive mice (see Supplementary material online, *Table S9*), consistent with previous report.^37^ Further, Cmpd17b has no marked impact on circulation leucocytes in our model (see Supplementary material online, *Table S9*), thus potentially exhibiting limited immune suppressive action with long-term administration. These observations suggest Cmpd17b lowers adverse structural and inflammatory proteins in LV potentially as a result of the improved resolution of inflammation and cardiovascular remodelling.

It is well documented that Ang II alters the structure and function of the vascular wall, mainly by causing deposition of ECM and increasing vascular stiffness^38,39^ consistent with our pentachromic staining results that demonstrated ECM organization was dysregulated in this study. In addition, our TA proteomic analysis demonstrated that structural proteins (Col18a1, Myh10) were up-regulated in the hypertensive mice contributing to vascular dysfunction. Further, our vascular ultrasound analysis demonstrated that hypertensive mice exhibit greater wall thickness and reduced distensibility, indicating vascular dysfunction consistent with studies using a similar dose and duration of Ang II.^17^ Increased stiffness and decreased compliance in the vascular wall are the main consequences of excessive vascular calcification and inflammation.^40^ Previous studies demonstrated that aortic calcification and inflammation were elevated in Ang II-induced hypertensive mice.^41^ Consistent with this, our Von Kossa stain analysis demonstrated that aortic calcification was elevated in hypertensive mice. This is consistent with the higher expression of calcium regulatory (Actn4) and inflammatory proteins (S100a1, Ptgis) in the TA proteome of hypertensive mice. Interestingly, Cmpd17b attenuated vascular dysfunction, calcification, and ECM disruption and decreased expression of calcium regulatory, structural, and inflammatory proteins in TA of hypertensive mice indicating a vascular compliance effect, consistent with observations of Cmpd17b vasoprotection in diabetic mice.^10^

In comparison with mouse tissue proteome, proteins associated with structural remodelling, inflammatory, calcium regulatory, and mitochondrial physiology were mildly dysregulated in Ang II-stimulated HASMCs and HCFs. Interestingly, these dysregulated processes were also evident in human tissue data sets. Cmpd17b rescued altered structural remodelling, mitochondrial physiology, inflammatory, and calcium regulatory proteins in Ang II-stimulated HASMCs and HCFs consistent with a restorative effect of Cmpd17b in mouse TA and LV proteome. Furthermore, Cmpd17b lowers the expression of genes involved in structural remodelling (*TIMP2*), inflammatory (*IL1β*), and pro-resolution regulation (*FPR1*, *FPR2*) and increases expression of genes related to mitochondrial physiology (*NDUFA7*) in Ang II-stimulated HASMCs suggesting the vasoprotective effect may be mediated by aortic smooth muscle cells. Our analysis revealed a direct correlation between blood pressure and cardiovascular parameters in hypertensive mice. However, hypertensive mice treated with Cmpd17b did not exhibit a significant correlation between blood pressure and cardiovascular parameters, consistent with its moderate reduction in blood pressure and greater efficacy in preventing cardiovascular damage (see Supplementary material online, *Figure S12*). These observations align with the findings from the cardiovascular proteome data, suggesting that the Cmpd17b effect is likely not dependent on blood pressure reduction but rather through its pro-resolving effect.

The pathological process of structural remodelling was associated with mitochondrial remodelling.^34^ Hypertension-induced mitochondrial structural abnormalities are often accompanied by alterations in mitochondrial metabolic and bioenergetic functions, including impaired respiration and increased production of reactive oxygen species (ROS).^42^ Increasing evidence supports the notion that mitochondrial abnormalities in hypertension may be caused by activation of the renin–angiotensin–aldosterone system.^43^ Previous reports have shown that Ang II-induced oxidative stress results in mitochondrial damage and dysfunction.^44^ It has been reported that increased myocardial oxygen consumption is associated with cardiac inefficiency in hypertensive patients and mice with diabetic cardiomyopathy.^45,46^ Changes in OCR tend to be dependent on the dose and the length of Ang II infusion.^47^ Our study demonstrated that the OCRs of cardiac Complexes 1, 2, and 4 were higher in vehicle-treated hypertensive mice, suggestive of reduced cardiac efficiency. Interestingly, Cmpd17b blunted the increased Complex 2 (succinate dehydrogenase) OCR, indicating Cmpd17b attenuated mitochondria dysregulation. Recent reports indicated that a high succinate concentration leads to reversed electron transfer (RET) from Complex 2 into Complex 1, associated with a higher rate of superoxide production.^48^ These observations may mean that Cmpd17b improves mitochondrial bioenergetics, particularly led by reduced Complex 2-induced ROS. Our proteomic data demonstrated that cardiovascular mitochondrial proteins (Fth1, Coq8a, and Ndufa7) were aberrant in vehicle-treated hypertensive mice, consistent with adverse mitochondrial remodelling. This is in line with previous observations that Ang II infusion in mice results in dysregulated expression of mitochondrial genes and proteins in LV and abdominal aorta.^31,32^ Cmpd17b normalized cardiac mitochondrial proteins (Taldo, Ndufb11) and aortic mitochondrial proteins (Naa15, Rpl26, and Ndufa7) in vehicle-treated hypertensive mice.

It is well-accepted that adverse structural remodelling and mitochondrial dysregulation contribute to end-organ damage in both humans^49^ and mice.^34^ In the current study, enrichment pathways and proteome remodelling associated with structural, inflammatory, and mitochondrial processes were dysregulated in LV and TA of Ang II-induced hypertensive mice. This is consistent with previous studies demonstrating that structural and inflammatory genes were up-regulated, and mitochondrial genes were altered in the human and mouse hypertensive heart^19,50^ and the abdominal aorta^18,51^ suggesting a pathological resemblance between progressive hypertension in humans and mice. Interestingly, chronic treatment with Cmpd17b shifts the cardiovascular proteome of Ang II-induced hypertensive mice towards normotensive levels, which indicates that Cmpd17b may also be capable of reducing adverse remodelling in hypertensive patients by improving structural and mitochondrial remodelling. NDUFA7 encodes a subunit of NADH:ubiquinone oxidoreductase (Complex 1 in mitochondrial respiratory chain), located in the inner mitochondrial membrane. This protein, involved in mitochondrial biogenesis, was dysregulated in hypertensive human and mouse tissue proteome data sets but was rescued by Cmpd17b. The beneficial effect of Cmpd17 was consistent with reduced *NDUFA7* expression in HASMCs stimulated with Ang II. These findings suggest that Cmpd17b may improve mitochondrial energetics as well as attenuate cardiovascular remodelling and function. In addition to the heart and aorta, Cmpd17b reduced kidney damage as reflected by lower fibrosis and improved kidney weight in vehicle-treated hypertensive mice, consistent with previous reports that an excess of Ang II caused renal impairment and kidney damage.^37^ We have demonstrated the preventive effects of Cmpd17b against Ang II-induced hypertension and end-organ damage as proof of concept. The exploration of Cmpd17b’s therapeutic potential necessitates further investigation, encompassing studies involving female mice and a comprehensive assessment of its protective effects on the progression of disease and kidney damage in hypertensive mice. Moreover, the execution of an intervention study with Cmpd17b in the future will contribute to the elucidation of its therapeutic efficacy, more closely aligned with clinical translation.

Overall, our comprehensive description of the mouse cardiac and aortic proteome reveals a select maladaptive and therapy-induced remodelling in proteins, networks, and pathways in hypertension. We found for the first time that the pro-resolving FPR agonist, Cmpd17b, normalizes structural remodelling and mitochondrial subproteomes in hypertensive mice. Supporting these findings, we further demonstrate a clear correlation with dysregulated expression of proteins in human hypertension suggesting FPR agonists might be efficacious in clinical settings. The novel FPR agonist Cmpd17b has an antihypertensive effect and prevents impaired cardiovascular function and remodelling in hypertensive mice, supporting the development of pro-resolution FPR-based therapies to treat complications of systemic hypertension. Thus, promoting the resolution of inflammation with FPR agonists may provide a new approach to targeting and potentially reversing hypertension-induced end-organ damage, supporting the development of FPR-based therapy to treat complications in systemic hypertension. Given the pronounced cardiac and vascular protective effect observed with a moderate reduction in blood pressure by Cmpd17b, a potential strategy that involves combining this agonist with clinical standard of care, such as an AT1 antagonist, could lead to synergistic improvement in attenuating hypertension-induced cardiovascular complications.

**Translational perspective**

Although existing therapies manage blood pressure, substantial end-organ damage remains. Here, we reveal the biased FPR agonist Cmpd17b exhibits moderate antihypertensive impact yet achieves robust attenuation of cardiovascular end-organ damage (likely secondary to enhanced resolution of inflammation). Comprehensive interrogation of the mouse cardiac and aortic proteome revealed marked hypertension-induced alterations in mitochondrial, calcium, and inflammation regulation, and in structural remodelling. Further, comparative analyses revealed the murine cardiac and aortic molecular maladaptation to hypertension correlates with that evident in humans; this is partially blunted by Cmpd17b in mice. FPR small-molecule agonists may offer a novel approach to treat hypertension-associated complications.

## Supplementary Material

cvae103_Supplementary_Data

## Data Availability

Data generated or analysed during this study are included in this published article (and its supplementary information files) or available from data repositories. Proteomic data (RAW and processed/search files) for each tissue region (TA, LV) and comparisons between human tissue are available from the ProteomeXchange Consortium via the PRIDE partner repository with the data set identifier PXD035034. For proteomic analyses, the Human Protein Atlas (https://www.proteinatlas.org/humanproteome/tissue) and functional enrichment annotations using g:Profiler (https://biit.cs.ut.ee/gprofiler/) were used. Further pathway enrichment map analysis was performed using Cytoscape (v3.7.1),^24^ Reactome,^24^ and DAVID functional annotation^24^ software. Protein–protein interaction networks were described using StringApp incorporated into Cytoscape (v3.7.1).^24^ Various human hypertensive data sets associated with TA^18^ or LV^19^ regions were compared. Hierarchical clustering was performed in Perseus using Euclidian distance and average linkage clustering, with missing values imputed at *z*-score 0. R was also used for data analysis and data visualization (ggplot2, ggpubr packages).
