# Supplementary material for: Novel formylpeptide receptor 1/2 agonist limits hypertension-induced cardiovascular damage
Source: Cardiovasc Res. 2024 Jun 16;120(11):1336–50. doi: 10.1093/cvr/cvae103 (PMC11416058; doi:10.1093/cvr/cvae103)
Supplement: cvae103_Supplementary_Data [file cvae103_supplementary_data.zip › Supplementary materials.docx]

**SUPPLEMENTARY MATERIALS**

**Novel formylpeptide receptor 1/2 agonist limits hypertension-induced cardiovascular damage**

**Authors**

Jaideep Singh, Kristy L Jackson, Haoyun Fang, Audrey Gumanti, Bethany Claridge, Feng Shii Tang, Helen Kiriazis, Ekaterina Salimova, Alex M Parker, Cameron Nowell, Owen L Woodman, David W Greening, Rebecca H Ritchie, Geoffrey A Head, Cheng Xue Qin

**LIST OF TABLES AND FIGURES**

[SUPPLEMENTARY FIGURES 16](#_Toc158202605)

1. [Supplementary Figure S1 Flow chart of animal use based on the CONSAERT template for preclinical studies. 16](#_Toc158202606)
2. [Supplementary Figure S2 Significantly changed structural, calcium regulatory and mitochondrial proteins in LV and TA of Ang II induced hypertensive mice. 17](#_Toc158202607)
3. [Supplementary Figure S3 Global proteome associated with vehicle and Cmpd17b treated normotensive and hypertensive TA and LV. 18](#_Toc158202608)
4. [Supplementary Figure S4 Significantly changed enrichment pathways in Ang II-stimulated HASMCs and HCFs. 19](#_Toc158202609)
5. [Supplementary Figure S5 Chronic effect of Cmpd17b or vehicle on change in cardiovascular parameters and locomotor activity over 28 days in hypertensive and normotensive mice. 20](#_Toc158202610)
6. [Supplementary Figure S6 Chronic effect of Cmpd17b or vehicle on cardiovascular parameters and locomotor activity over 28 days in hypertensive and normotensive mice. 22](#_Toc158202611)
7. [Supplementary Figure S7 Acute effect of losartan and Ang II on MAP in Ang II-induced hypertensive and normotensive mice treated with vehicle or Cmpd17b. 24](#_Toc158202612)
8. [Supplementary Figure S8 Chronic Cmpd17b treatment blunts renal mass but not glomerulosclerosis index in Ang II-induced hypertensive mice. 26](#_Toc158202613)
9. [Supplementary Figure S9 Chronic Cmpd17b treatment blunts collagen and calcium deposition but not elastin percentage in Ang II-induced hypertensive mice. 27](#_Toc158202614)
10. [Supplementary Figure S10 Commonly identified enrichment pathways in human hypertension and Cmpd17b-treated hypertensive mice. 28](#_Toc158202615)
11. [Supplementary Figure S11 Significantly changed structural, calcium regulatory and mitochondrial proteins in heart and aorta of human hypertensive datasets. 29](#_Toc158202616)
12. [Supplementary Figure S12 Correlation of blood pressure with cardiovascular remodelling and function in Ang II-induced hypertensive mice treated with vehicle and Cmpd17b. 31](#_Toc158202617)

[SUPPLEMENTARY TABLES 32](#_Toc158202618)

1. [Supplementary Table S1 Effect of Cmpd17b or vehicle on cardiovascular parameters in hypertensive and normotensive mice. 32](#_Toc158202619)
2. [Supplementary Table S2 Common and significant proteins in LV and TA of mouse and human hypertension. 34](#_Toc158202620)
3. [Supplementary Table S3 Full name of all the listed proteins in proteome analysis. 35](#_Toc158202621)
4. [Supplementary Table S4 B-mode echocardiography analysis of LV in hypertensive and normotensive mice treated with vehicle or Cmpd17b. 39](#_Toc158202622)
5. [Supplementary Table S5 M-mode echocardiography analysis of LV in hypertensive and normotensive mice treated with vehicle or Cmpd17b. 40](#_Toc158202623)
6. [Supplementary Table S6 Body weight-normalised organ weights in hypertensive and normotensive mice treated with Cmpd17b or vehicle. 41](#_Toc158202624)
7. [Supplementary Table S7 Carotid artery characteristics assessed from ultrasound imaging in hypertensive and normotensive mice treated with Cmpd17b or vehicle. 42](#_Toc158202625)
8. [Supplementary Table S8 Common and significant proteins in LV and aorta of mouse hypertensive treated with Cmpd17b and human hypertensive datasets. 43](#_Toc158202626)
9. [Supplementary Table S9 Haematological differential blood cell counts in vehicle or Cmpd17b-treated hypertensive and normotensive mice. 44](#_Toc158202627)
10. [Supplementary Table S10 List of gene primers 45](#_Toc158202628)
11. [Supplementary Table S11 TA tissue quantitative proteomics for groups WT-Veh, WT-Cmpd17b, Hypertensive-Veh, and Hypertensive-Cmpd17b. 46](#_Toc158202629)
12. [Supplementary Table S12 LV tissue quantitative proteomics for groups WT-Veh, WT-Cmpd17b, Hypertensive-Veh, and Hypertensive-Cmpd17b. 46](#_Toc158202630)
13. [Supplementary Table S13 T-Test analysis of TA tissue proteomics of WT, Cmpd17b and NTN groups. Red highlights p<0.05 46](#_Toc158202631)
14. [Supplementary Table S14 Functional enrichment of proteins from TA tissue proteomics from HTN_Veh vs WT_Veh group. Increased (high) enrichment in HTN_Veh group. 46](#_Toc158202632)
15. [Supplementary Table S15 T-Test analysis of LV tissue proteomics of WT, Cmpd17b and NTN groups. Red highlights p<0.05 46](#_Toc158202633)
16. [Supplementary Table S16 Functional enrichment of proteins from LV tissue proteomics from HTN_Veh vs WT_Veh group. Increased (high) enrichment in HTN_Veh group. 46](#_Toc158202634)
17. [Supplementary Table S17 Functional enrichment of proteins from TA tissue proteomics from HTN_Cmpd17b vs HTN_Veh group. Increased (high) enrichment in HTN_Cmpd17b group. 46](#_Toc158202635)
18. [Supplementary Table S18 Functional enrichment of proteins from LV tissue proteomics from HTN_Cmpd17b vs HTN_Veh group. Increased (high) enrichment in HTN_Cmpd17b group. 46](#_Toc158202636)
19. [Supplementary Table S19 Proteins identified in LV and TA tissue of hypertensive mice+Veh but not in normotensive mice+Veh. 46](#_Toc158202637)
20. [Supplementary Table S20 Differential proteins of HASM cells proteomics from Ang II_Veh vs control_Veh and Ang II_Cmpd17b vs AngII_Veh group. 47](#_Toc158202638)
21. [Supplementary Table S21 Functional enrichment of proteins from HASM cells proteomics from Ang II_Veh vs control_Veh and Ang II_Cmpd17b vs AngII_Veh group. 47](#_Toc158202639)
22. [Supplementary Table S22 Differential proteins of HCF cells proteomics from Ang II_Veh vs control_Veh and Ang II_Cmpd17b vs AngII_Veh group. 47](#_Toc158202640)
23. [Supplementary Table S23. Functional enrichment of proteins from HCF cells proteomics from Ang II_Veh vs control_Veh and Ang II_Cmpd17b vs AngII_Veh group. 47](#_Toc158202641)
24. [Supplementary Table S24. Total identified proteins of HASM cells proteomics from Ang II_Veh vs control_Veh and Ang II_Cmpd17b vs AngII_Veh group. 47](#_Toc158202642)
25. [Supplementary Table S25. Total identified proteins of HCF cells proteomics from Ang II_Veh vs control_Veh and Ang II_Cmpd17b vs AngII_Veh group. 47](#_Toc158202643)
26. **MATERIALS AND METHODS**

**1.1 Animals**

Twelve-week-old male C57BL/6J mice were housed individually in a room with 12:12 h light-dark cycle (1 a.m.–1 p.m. light) and allowed access *ad libitum* to water and mouse chow (Specialty Feeds, Glen Forrest, Western Australia, 19% protein, 5% fat, 5% fibre, 0.2% sodium). All experiments were conducted in accordance with the Australian Code of Practice and the National Health and Medical Research Council and ARRIVE guidelines^1^.

**1.2 Telemetry probe implantation**

Blood pressure (BP) telemetry transmitters (model TA11PA-C10; Data Sciences International, St Paul, Minnesota, USA) were implanted under isoflurane open circuit anesthesia (4% induction and 1.5–2% maintenance; Forthane, Abbott, Botany, Australia). The catheter of the telemetry device was inserted into the left carotid artery and the transmitter probe was positioned subcutaneously along the right flank. Post-operative analgesia was provided by subcutaneous (SC) administration of 5 mg/kg carprofen (Pfizer Australia Pty Ltd., West Ryde, NSW, Australia)^2, 3^.

1. **Angiotensin II (Ang II)-induced hypertension model**

At 15-weeks of age, mice were again anesthetized with isoflurane to subcutaneously implant an osmotic minipump (2004, Alzet; Cupertino, CA, USA) containing either Ang II (0.7 mg/kg/day; Auspep, Tullamarine) or saline (Pfizer; New York City, NY, USA) at an infusion rate of 6 µl/day over the 28-day treatment period. The minipumps were inserted via a small incision made between the scapulas and positioned subcutaneously on the left flank. Both carprofen and bupivacaine (5 mg/kg) were SC administered to the mouse as described previously^4^.

**1.4 Intraperitoneal injection of compound17b (Cmpd17b)**

Mice implanted with saline or Ang II minipumps were treated with either Cmpd17b (50 mg/kg/day; i.p.; Anthem Biosciences Bengaluru, Karnataka, India) or vehicle once daily at ~9:00 am for 28 days. Vehicle was comprised of 0.8% Tween 80 (Sigma-Aldrich, St. Louise, USA), 10% DMSO (AMRESCO® Solon, Ohio, USA) and saline (Pfizer; New York City; NY; USA). Cmpd17b was dissolved in the vehicle and sonicated for 15 minutes. The dose, delivery method and duration of Cmpd17b dissolved in the vehicle has been used in previous studies involving mouse models of myocardial infarction and diabetes with no adverse effects observed^5, 6^.

**1.5 Cardiovascular markers and locomotor activity measurements**

After a 10-day recovery period from telemetry surgery, 24-hour continuous recording of systolic (SAP), diastolic (DAP), and calculated mean arterial pressure (MAP), heart rate (HR), and locomotor activity were measured prior to surgery (baseline) and then re-measured after 3, 5, 7, 12, 15, 19, 22, 26 and 28 days in conscious, unrestrained mice. The recordings were sampled at 1,000 Hz using an analogue-to-digital data acquisition card (PCI-8024e; National Instruments; Austin, TX, USA) as described previously^7^.

**1.6 Acute BP response to Cmpd17b in conscious mice**

To determine the acute MAP response to Cmpd17b, the blood pressure in the 40-60 minutes following injection was compared with the 60 minutes prior to injection on days 2 and 28. The acute MAP response to Cmpd17b was determined as 10 minute averages across the 60 minutes before and 40-60 minutes after injection.

***Acute pressor response to Ang II***

Acute Ang II challenge was used to assess the BP response to Ang II. Ang II (0.2 mg/kg; i.p.; Auspep; Tullamarine, VIC, Australia) was administered during the last week of the experiment. The challenge was performed during the light/inactive period of the mice. Radiotelemetry-implanted mice were injected daily with vehicle or Cmpd17b for 4 weeks. During week 4, a stable resting baseline of BP was recorded, and mice were administered with saline. After a stable 3-minute baseline had been re-established, mice were administered Ang II (0.2mg/kg; i.p.). MAP across the 3 minutes before and after the Ang II injections were compared. The acute MAP response to Ang II was expressed as 30-second averages across the 3-minute pre- and post-injection.

***Acute depressor response to AT1 receptor antagonist losartan***

Acute losartan challenge was used to assess the effect of Cmpd17b treatment on the contribution of the renin-angiotensin system (RAS) to BP maintenance in Ang II-induced hypertensive and normotensive mice. The AT1 receptor antagonist losartan (10 mg/kg; i.p.; Auspep; Tullamarine, VIC, Australia) was administered during the last week of the treatment period. The challenge was performed during the dark/active period of the mice. Radiotelemetry-implanted mice were injected daily with vehicle or Cmpd17b for 4 weeks. During week 4, a stable 60-minute resting baseline of BP was recorded, and mice were administered saline. After a stable 60-minute baseline had been re-established, mice were administered losartan (10 mg/kg i.p.). MAP across the 60 minutes before and after the losartan injection was compared. The acute MAP response to losartan was expressed as 10 minute averages across the 60 minutes pre- and post-injection.

**1.7 Spectral analysis of mid-frequency blood pressure variability**

Spectral analysis of mid-frequency (0.3-0.5 Hz) MAP variability was used to determine the effect of Cmpd17b on sympathetic activity. BP readings at baseline, one-week post treatment and four-weeks post treatment were analysed in a program written in LABview (National Instruments, USA) for data processing to produce power spectra. Four replicates of 1 hour readings were analysed for each animal. All data segments analysed were taken during the active/dark period. Within 1 hour readings the area under the curve for HR and MAP was calculated by generating power spectra of at least 4 overlapping segments utilizing a Fast Fourier transformation as previously described^8, 9^. A reading was considered acceptable if the coherence value between MAP and HR was ≥0.4.

**1.8 Tissue collection**

Animals were euthanized with pentobarbital sodium (100 mg/kg, i.p.). Blood was collected into an ethylenediaminetetraacetic acid (EDTA) tube (Microvette, SARSTEDT, Germany) by performing cardiac puncture. Heart, kidneys, lungs, liver, spleen, adrenal glands and vessels were washed and collected. All organ weights except adrenal glands and vessels were recorded. The heart was further dissected into left ventricle (LV), right ventricle (RV) and left and right atrium and the weights were recorded. One segment of LV was placed in a cassette and immersed in 10% formaldehyde for histology and other segment of LV and thoracic aorta was placed in a 2 ml Eppendorf and snap frozen in liquid nitrogen for proteomics analysis. Kidneys were placed in a cassette and immersed in formaldehyde for histology. The abdominal aortas were placed in cassettes in calcium-free Krebs solution followed by fixing in formaldehyde. All these formaldehyde fixed sections were dehydrated, embedded, viewed at 40X magnification, and photographed.

**1.9 Cardiac, renal and vascular remodelling**

All staining and imaging were performed by the Monash Histology Platform. The whole slide was scanned, and raw ScanScope virtual slide files were provided by the Monash histology platform. Aperio ImageScope (Leica Microsystems Pty. Ltd.) software was used to extract the whole image. All image analysis was carried out using custom written macros (code available on request) in the Fiji distribution of Image J software (version 1.53c, National Institute of Health, USA)^10^. LV ^11^, kidney^12^, and vessel^13^ sections were stained with picrosirius red to detect collagen deposition. The percentage area of red-stained collagen was quantified by a color deconvolution and thresholding in Image J software (version 1.53c, National Institute of Health, USA). LV sections were stained with hematoxylin and eosin to measure cardiomyocyte area and width using a semi-automated macro in Image J software (version 1.53c, National Institute of Health, USA)^11^. Verhoeff–van Gieson (VVG) stain identified elastin^13^, Pentachrome stain indicated mucin and fibrin^14^, and the Von Kossa and Alizarin Red stain indicated calcium deposition^15, 16^ in vessels. Stained colors relevant to elastin, mucin, fibrin, and calcium area in the vessel were extracted using trained Ilastik models^17^ in Image J software (version 1.53c, National Institute of Health, USA). These extracted regions were then used to calculate area coverage and percentages. The tissue and stain area were averaged in Excel (Version 2019, Microsoft). For renal histology, kidney sections (3 µm) were stained with periodic acid–Schiff (PAS)^18^. The degree of sclerosis in each PAS-stained glomerulus was subjectively graded, in a blinded manner, on a scale of 0–4: grade 0, normal; grade 1, sclerotic area up to 25% (minimal); Grade 2, sclerotic area 25–50% (moderate); Grade 3, sclerotic area 50–70% (moderate–severe) and Grade 4, sclerotic area 75–100% (severe). The glomerulosclerosis index (GSI) histology score was then calculated using the following formula: GSI = (1 × *n*1) + (2 × *n*2) + (3 × *n*3) + (4 × *n*4)/*n*0 + *n*1 + *n*2 + *n*3 + *n*4, where *n*x is the number of glomeruli in each grade of glomerulosclerosis.

**1.10 Ultrasound imaging**

Cardiac^19^ and vascular function^20^ were evaluated by ultrasound imaging using a Vevo 2100 High-Resolution Imaging System (Visual Sonics Inc., Canada). In brief, two-dimensional, long-axis echocardiography and M-mode echocardiography was performed to assessed LV systolic function. Ultrasound imaging of the carotid artery was performed to assessed vascular function.

**1.11 Differential cell count**

Differential cell count was used to assess the involvement of the immune system systemically. A small aliquot of blood (20 µl) was diluted in 120 µl cell pack (Sysmex, Japan) used for differential cell count and blood cell count data was acquired by XS-1000i automated hematology analyzer (Sysmex, Japan).

**1.12 Mitochondrial respiration assessment in frozen samples by sea horse assay**^21^

***Tissue homogenization***

4 mg of frozen LV tissues were thawed, minced, and homogenized in 0.2 ml of mitochondrial assay solution (MAS) buffer (70 mM sucrose, 220 mM mannitol, 5 mM KH_2_PO_4_, 5 mM MgCl_2_, 1 mM EGTA, 2 mM HEPES pH 7.4). LV tissues were incubated in an incubator at 37°C for 30 mins. All homogenates were centrifuged at 1,000 *g* for 10 min at 4°C; then, the supernatant was collected. Protein concentration was determined by bicinchoninic acid **(**BCA, Thermo Fisher).

***Plate loading and substrate injection***

LV homogenates were loaded into a Seahorse XF96 microplate reader in 20 μl of MAS. The loaded plate was centrifuged at 2,000 g for 5 min at 4°C (without brake) and an additional 140 μl of MAS containing cytochrome c (10 μg/ml, final concentration) was carefully added to each well to avoid disrupting the bottom of the plate. Substrate injection was as follows: NADH (1 mM), or 5 mM succinate + rotenone (5 mM + 2 μM) was injected at port A; rotenone + antimycin A (2 μM + 4 μM) at port B; TMPD + ascorbic acid (0.5 mM + 1 mM) at port C; and azide (50 mM) at port D. These conditions allow for the determination of the respiratory capacity of mitochondria through Complex 1, Complex 2, and Complex 4.

***Mitochondrial respiration analysis***

Wave software (Agilent) was used to export OCR rates normalized by protein. Complex 1, 2, and 4‐dependent respiration was calculated by subtracting OCR values (maximum-minimum).

**1.13** **Quantitative tissue-based proteomics analysis**

***Sample homogenisation, protein reduction, alkylation, and digestion***

Left ventricle (LV, 5 mg) and thoracic aorta (TA, 2 mg) samples were harvested from saline infused vehicle treated mice (n=5), saline infused Cmpd17b treated mice (n=5), Ang II infused vehicle treated mice (n=5), Ang II infused Cmpd17b treated mice (n=5) and lysed on ice in lysis buffer (8M urea in 50 mM HEPES, pH 8.0) with protease and phosphatase inhibitor (Halt, Life Technologies, #78442) and extracted by tip-probe sonication and quantified by microBCA (Life Technologies, #23235). Lysates (10 µg protein) were normalized in 50 µL lysis buffer. Samples reduced (10 mM dithiothreitol, DTT) for 1 hr at 25°C, alkylated (20 mM iodoacetamide) for 30 min at 25°C in the dark and quenched with additional DTT (10mM) before the Sera‐Mag-based workflow^22, 23^. Magnetic bead slurry was prepared by mixing SpeedBeads™ magnetic carboxylate modified particles (Cytiva, 65152105050250, 45152105050250) at 1:1 (v:v) ratio, washing with MS-grade water and reconstituted to a final concentration of 100 µg/µL. The beads were added at 10:1 beads-to-protein ratio and ethanol (EA043, ChemSupply) added to a final concentration of 50% (v/v). Protein-bound magnetics beads were washed three times with 200 µL of 80% ethanol and reconstituted in 50 µL of 50 mM triethylamonium bicarbonate (TEAB) pH 8.0. Protein digestion was performed with Lysyl Endopeptidase (enzyme:substrate 1:100, 125-05061, Wako Pure Chemical Industries) and trypsin (enzyme:substrate 1:50, Promega V5113) overnight at 37°C with agitation (1,000 rpm). The peptide mixture was acidified to a final concentration of 2% formic acid and centrifuged at 20,000*g* for 1 min. The peptide digests were kept frozen at ^-^80°C and dried by vacuum centrifugation, reconstituted in 0.07% trifluoroacetic acid, and quantified by Fluorometric Peptide Assay (Thermo Scientific, 23290) as per manufacturer’s instructions.

***NanoLC and mass spectrometry***

Spectra acquired in data dependent acquisition on an Q Exactive HF-X benchtop Orbitrap mass spectrometer coupled to an UltiMate™ NCS-3500RS nano-HPLC (Thermo Fisher Scientific) as previously described^24, 25^. Peptides (360 ng) were loaded (Acclaim PepMap100 C18 3 μm beads with 100 Å pore-size, Thermo Fisher Scientific) and separated (1.9 µm particle size C18, 120Å, 0.075 × 200 mm, Nikkyo Technos Co. Ltd) with a gradient of 2–28% acetonitrile containing 0.1% formic acid over 95 mins followed by 28-80% from 95-98 mins at 300 nL min-1 at 55°C (butterfly portfolio heater, Phoenix S&T). An MS1 scan was acquired from 350–1,650 m/z (60,000 resolution, 3 × 10^6^ automatic gain control (AGC), 128 msec injection time) followed by MS/MS data-dependent acquisition (top 25) with collision-induced dissociation and detection in the ion trap (30,000 resolution, 1 ×10^5^ AGC, 60 msec injection time, 28% normalized collision energy, 1.3 m/z quadrupole isolation width). Unassigned precursor ions charge states and slightly charged species (were rejected and peptide match disabled. Selected sequenced ions were dynamically excluded for 30 sec). Data was acquired using Xcalibur (v4.5, Thermo Fisher Scientific). For spectral library generation and high-pH fractionation, anhydrous peptide digests from LV (pooled) and TA (pooled) were individually reconstituted in 25 mM ammonium formate, pH 10 for high pH reversed phase (RP) microscale fractionation. In-house X-RPS Stagetips (#66886-U, Sigma) were used for peptide binding and peptides eluted using acetonitrile (2-50%, v/v) in 25 mM ammonium formate, pH 10. A total of 8 fractions from each tissue region were lyophilised by speedVac. Peptide samples were reconstituted in 0.07% triflouroacetic acid (TFA) analysed in single shot proteomics as described. For tissue-based analyses a list of samples and RAW data (including spectral libraries) is available in ProteomeXchange Consortium via the PRIDE partner repository; #PXD035034.

**1.14** **Cell Culture**

Human aortic smooth muscle cells (HASMCs) were obtained from American Type Culture Collection (ATCC Manassas, VA, USA)^26^ and cultured in basal vascular cell media from ATCC (Manassas, VA, USA) containing 5% Fetal bovine serum (FBS) in a humidified incubator at 37C with 5% CO2. Cells from passages 5 to 8 were used for the experiments. 50% confluent HASMCs were seeded at 2 × 10^5^ cells per ml in a 6-well plate. These cells were platted in duplicate wells and grown for 3 days. Treatment was performed when cells were approximately 80% confluent. Briefly, HASMCs were exposed to Ang II (300 nM) treated with Cmpd17b (10 μM) or veh (0.1% DMSO), 2 h before Ang II stimulation as the prevention approach or 3 h post-Ang II treatment as the intervention approach. At the end of 6 h, cells were collected to measure gene expression using quantitative real-time polymerase chain reaction^27^. A separate cohort of cells were collected at 24 h for proteomics analysis, as described^28^.

Human primary cardiac fibroblasts (HCFs) were cultured as previously described^29^. Human ventricle cardiac fibroblasts obtained from Lonza (CC-2904, batch 20TL356511; ventricle) and cultured on flasks (coated with 1% gelatin (bovine skin Type B, Sigma-Aldrich, 9000-70-8) overnight) in media consisting of 50% cardiac fibroblast growth medium (Lonza, CC-4526), 45% DMEM/F12 (Gibco, Invitrogen, 11320033), 5% 0.22μm filtered foetal calf serum (Gibco, Invitrogen, 10099141), and penicillin-streptomycin (Gibco, Invitrogen, 15140122) at 37°C, 5% CO2. Cells were passaged 1:3 by surface area and used at passage 6. Fibroblasts were plated at confluency on plastic plates coated with 1% gelatin and left to attach overnight, after which media was replaced with Ang II (1 μM) or saline. After 3 hours, cells were treated with Cmpd17b (10 μM) or veh (0.1% DMSO). At the end of 24 h, cells were collected to perform proteomics analysis.

Sample preparation was performed as above, with 10 µg protein normalised in 50 µl volume of 50 mM HEPES and 1% SDS (pH 8). Spectra were acquired in data independent acquisition on a Q Exactive HF-X benchtop Orbitrap mass spectrometer coupled to an UltiMate™ NCS-3500RS nano-HPLC (Thermo Fisher Scientific) as described^28^. MS-based cellular proteomics data is deposited to the ProteomeXchange Consortium via the MASSive partner repository and available via MASSive with identifier (MSV000093917).

**1.15 Data processing and bioinformatics**

For data-dependent acquisition (DDA) tissue proteomic data analysis, identification and quantification of peptides was performed using MaxQuant (v1.6.14.0)^30^ and Andromeda^31^ as described^25, 32^. For each tissue region (LV or TA) tandem mass spectra were searched against *Mus musculus* (mouse) reference proteome (UP000000589; 55,398) supplemented with common contaminants and generated spectral library. Search parameters were as follows: carbamidomethylated cysteine as fixed modification, oxidation of methionine and N-terminal protein acetylation as variable modifications, trypsin/P as proteolytic enzyme with ≤ 2 missed cleavage sites, search tolerance 7 ppm, fragment ion mass tolerance 0.15 Da; minimum peptide length was defined at 6, <1% false discovery rate on peptide spectrum match with target-decoy approach at peptide and protein levels, match between runs (MBR) selected, and label free quantification (LFQ) algorithm employed. Contaminants, and reverse identification were excluded from further data analysis. High confident protein identification required more than one unique or razor peptides per protein group.

For data-independent acquisition (DIA) cellular proteomic data analysis, DIA-NN (v1.8) software suite^33^ was used for the identification and quantification of peptides as described^28^. For each cell model, acquired spectra were searched independently against *Homo sapiens* (human) reference proteome (UP000005640; 82,685) with library-free search/library generation mode and match between runs (MBR) enabled. Trypsin/P digestion was selected with maximum 1 missed cleavage. Peptide length range was set to 7-30 with N-term methionine excision and cysteine carbamidomethylation included as a fixed modification. Precursor charge range was set to 1-4. Precursor and fragment in m/z range was set to 300-1800 and 200-1800, respectively, with 1% FDR set for precursor identifications.

Data analysis was performed using Perseus of the MaxQuant computational platform^34^ and R programming language. To include high confidence and quantitative protein groups, we included protein groups identified in at least 70% of at least one group. By using Perseus software, protein intensities were log2 transformed and subjected to PCA with missing values imputed from normal distribution (width 0.3, downshift 1.8). Stringent data quality inclusion was applied with 70% protein group quantification for proteins within a group (tissue and cell analyses). Student’s t-tests were commenced with permutation-based multiple testing (q-val) enabled in Perseus software suite (default settings: FDR 0.05, 250 randomizations). Hierarchical clustering was performed using Euclidian distance and average linkage clustering. By using R package ggplot2, the violin plot of the protein intensity distribution and the boxplot of coefficient of variations per sample group were visualised. The volcano plot of Student’s t-test p-value versus log2 fold change was generated. g:Profiler and R package ClusterProfiler were utilized for functional enrichment and network/pathway analysis, significance p<0.05 as described previously^24, 35^.

**Gene expression**

Total RNA was isolated from cells using Trizol Reagent (Life Technologies, CA, USA)^27^ and reverse-transcribed (Applied Biosystems), as per the manufacturer’s instructions. Relative expression was determined using polymerase chain reaction using SYBR Green chemistry (Applied Biosystems), and primers generated from human sequences in GenBank (Supplementary Table S10). Quantitative analysis was performed using the QuantStudio7 Flex system (Applied Biosystems), using the 2^–ΔΔ Ct^ method to detect fold differences relative to the defined comparison group^36^.

1. SUPPLEMENTARY FIGURES

### *Supplementary Figure S1 Flow chart of animal use based on the CONSAERT template for preclinical studies.*


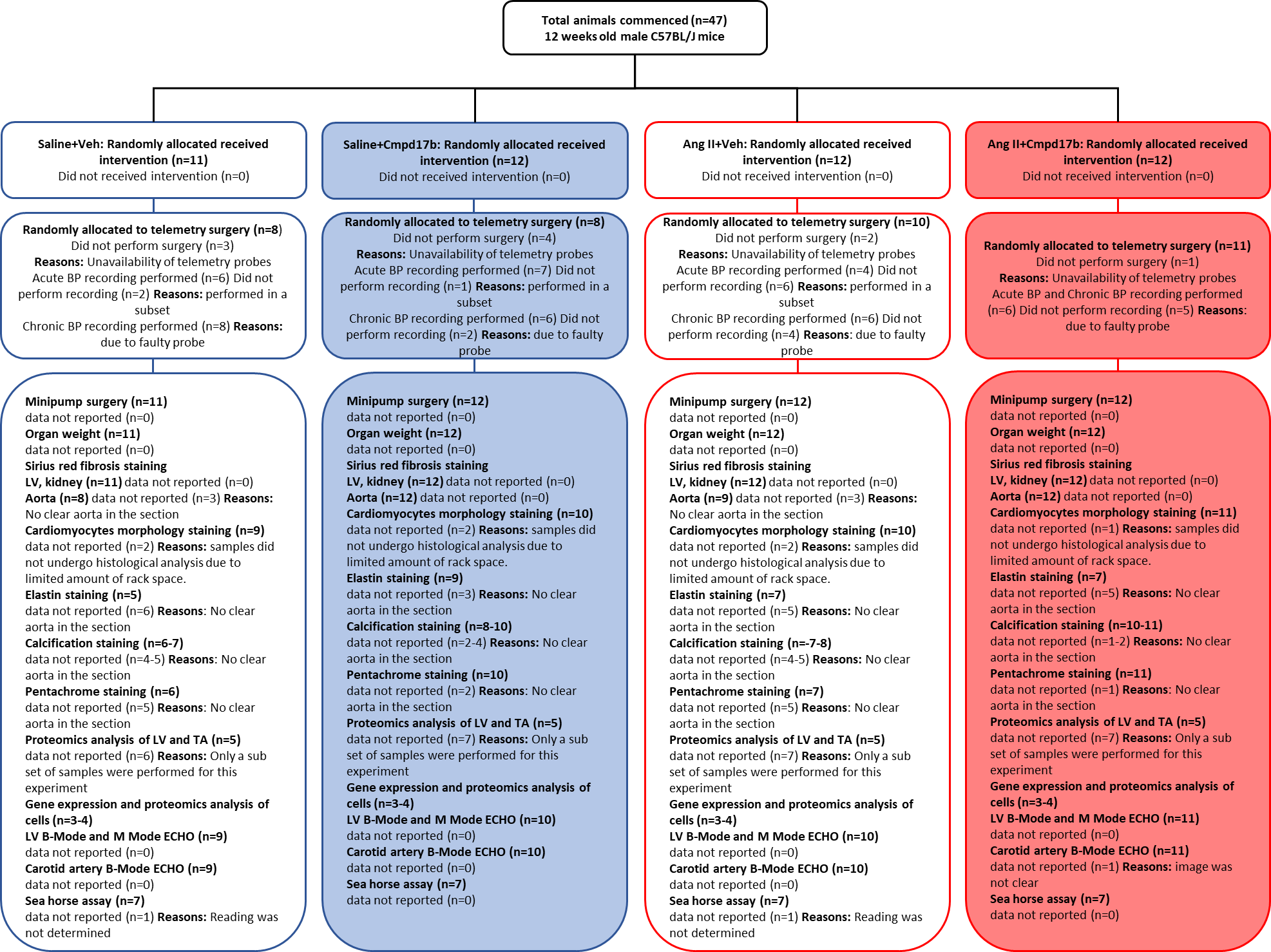


**
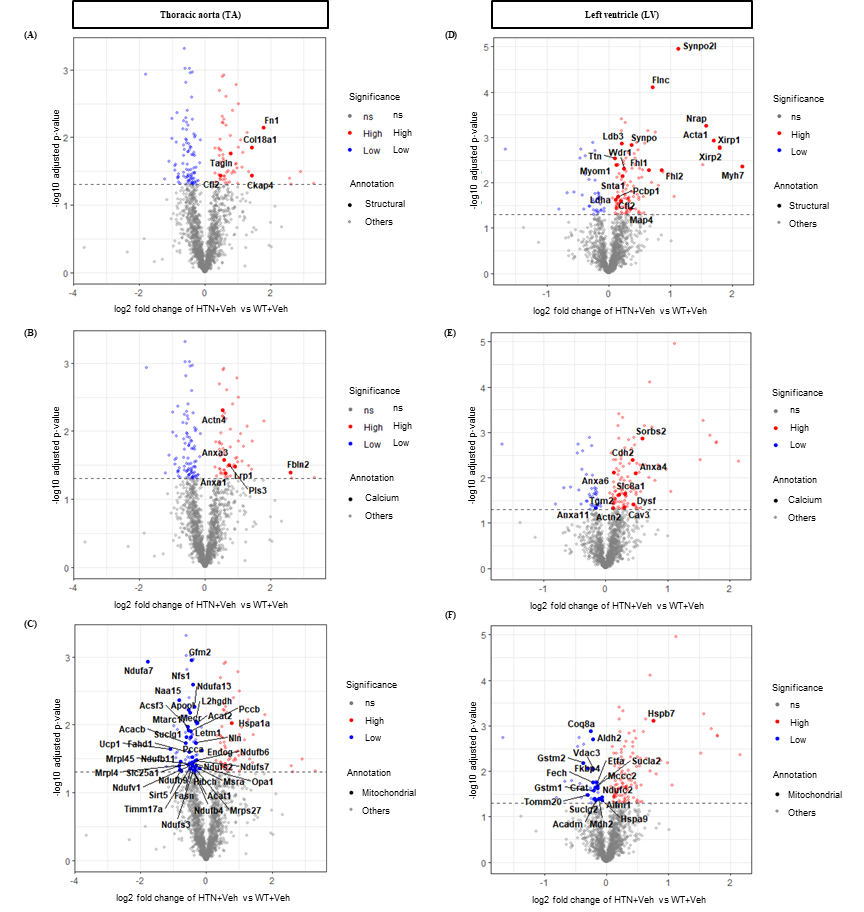
**

### *Supplementary Figure S2 Significantly changed structural, calcium regulatory and mitochondrial proteins in LV and TA of Ang II induced hypertensive mice.*

Volcano plots represent the log2fold change of structural, calcium regulatory and mitochondrial proteins in TA and LV (A-F). All the proteins displayed in the graph were significantly differentiated (*P<0.05*) in vehicle (veh)-treated hypertensive (HTN) mice (HTN+Veh) compared with vehicle-treated normotensive (wildtype-WT) mice (WT+Veh).

**
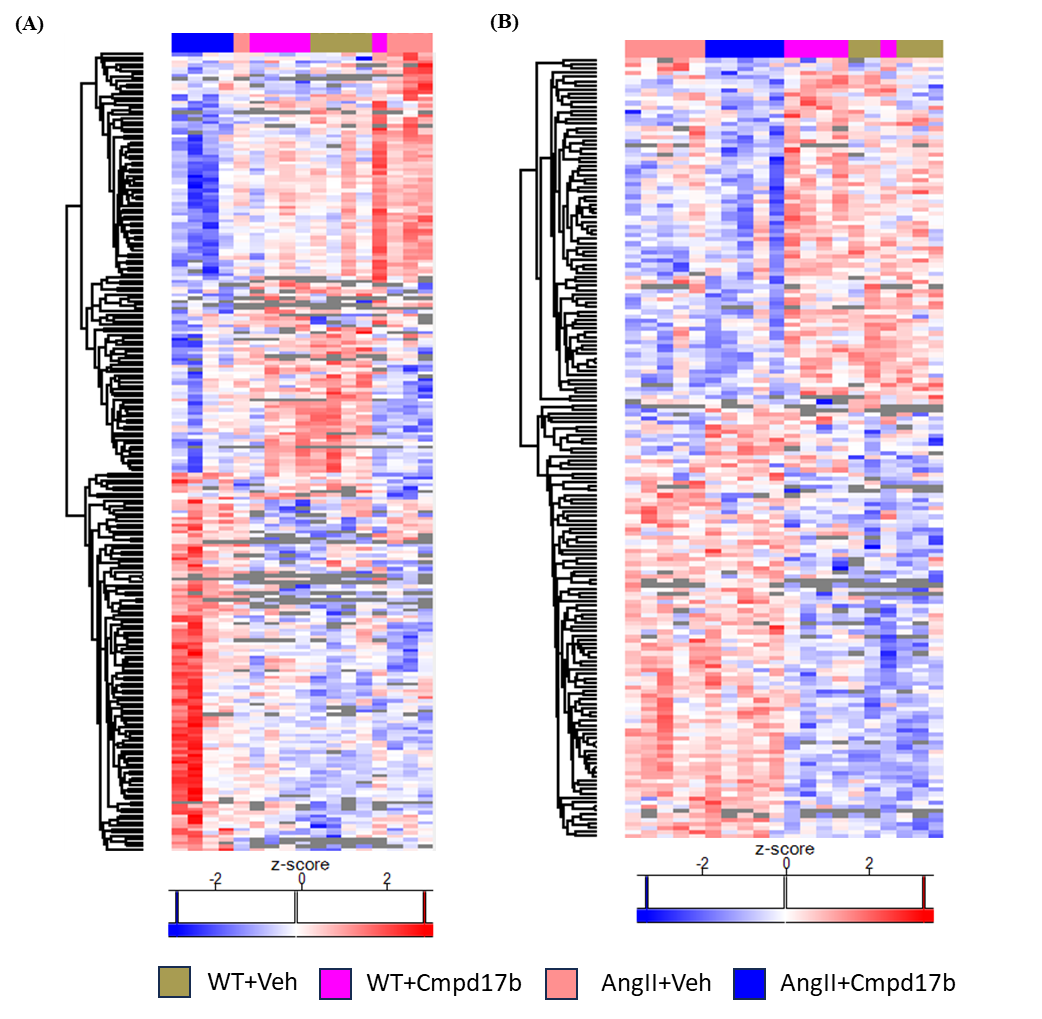
**

### *Supplementary Figure S3 Global proteome associated with vehicle and Cmpd17b treated normotensive and hypertensive TA and LV.*

Heatmap demonstrated global proteome of TA (A) and LV (B) comparing proteins in vehicle-treated normotensive (WT+Veh) mice, vehicle-treated hypertensive mice (AngII+Veh), Cmpd17b-treated normotensive (WT+Cmpd17b) mice, Cmpd17b-treated hypertensive mice (AngII+Cmpd17b).


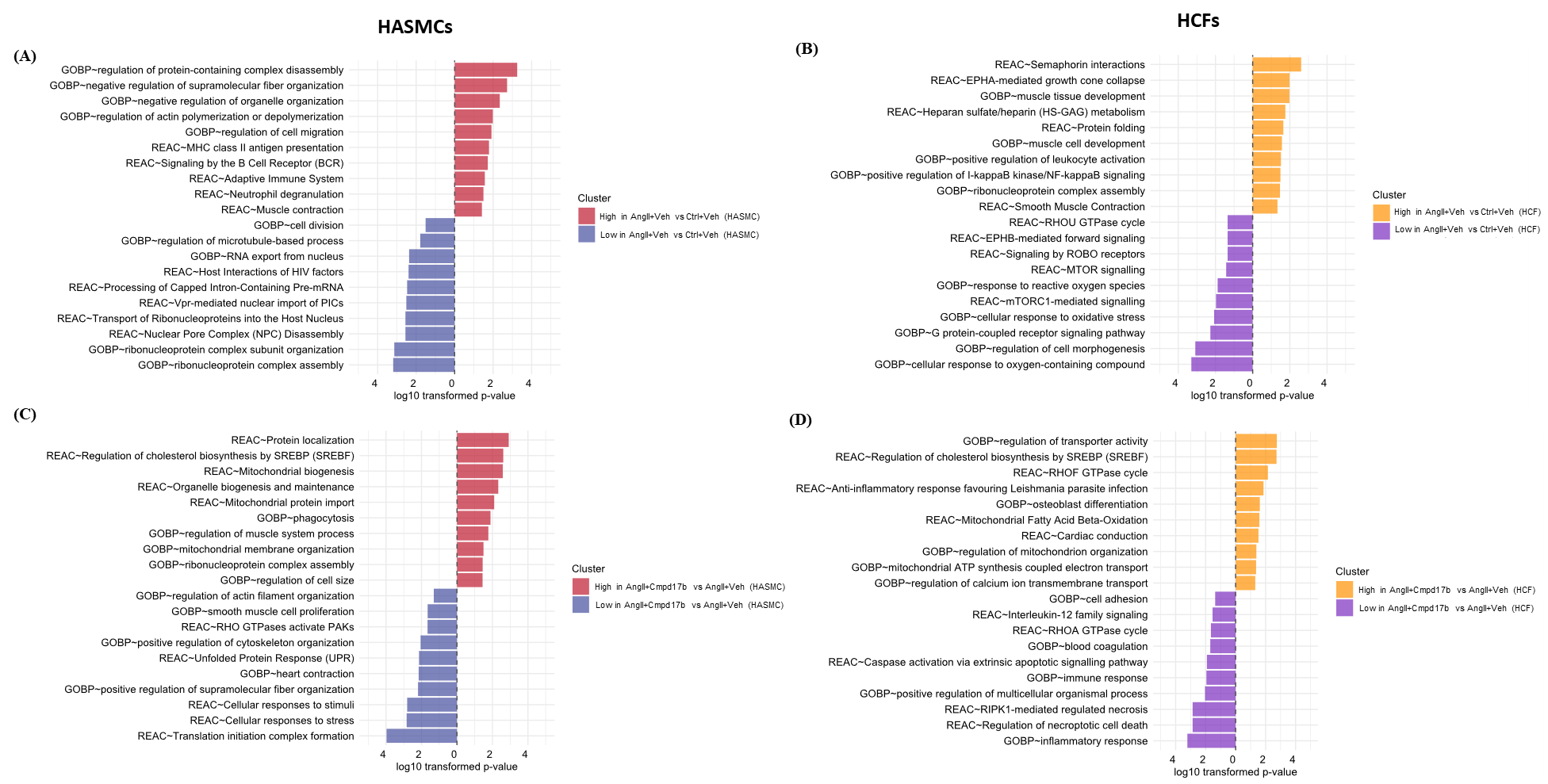


### *Supplementary Figure S4 Significantly changed enrichment pathways in Ang II-stimulated HASMCs and HCFs.*

Upregulated (red) and downregulated (blue) enrichment processes in AngII-stimulated HASMCs and HCFs treated with Veh compared to Ctrl (A-B) and Cmpd17b (C-D). Veh: vehicle, AngII: angiotensinII, HCFs: human cardiac fibroblasts, HASMCs: human aortic smooth muscle cells, Ctrl: control.


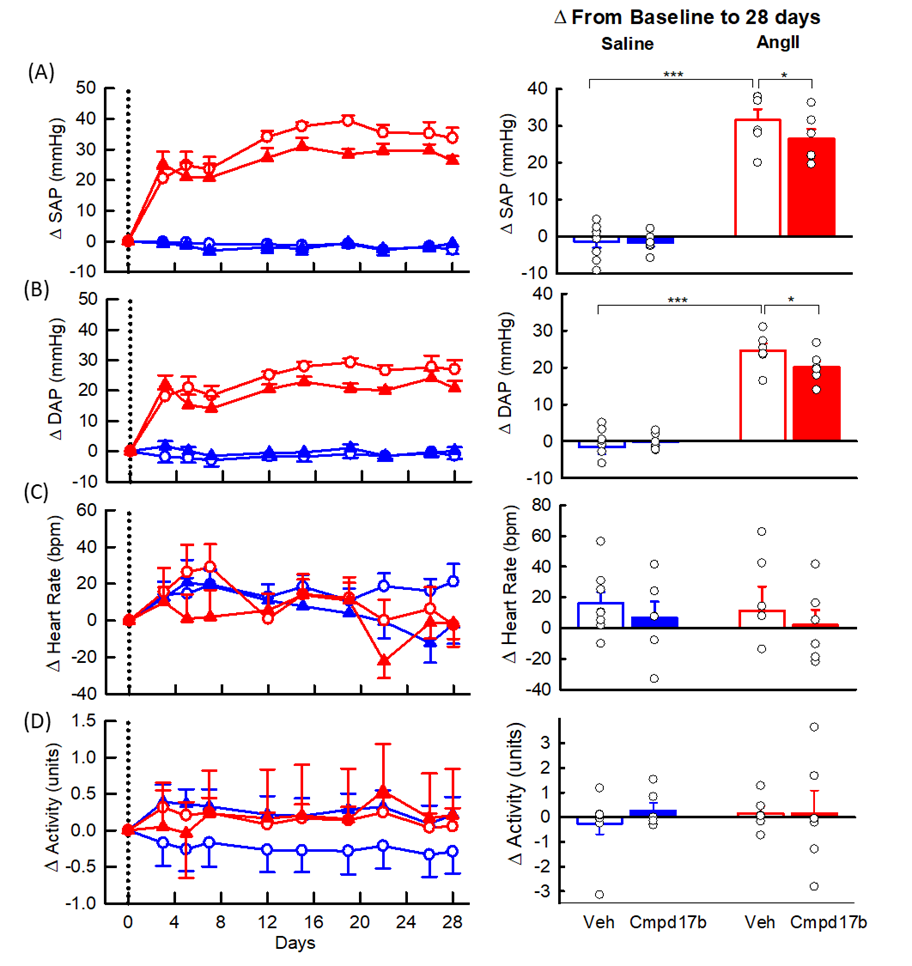


### *Supplementary Figure S5 Chronic effect of Cmpd17b or vehicle on change in cardiovascular parameters and locomotor activity over 28 days in hypertensive and normotensive mice.*

Line graphs (left) show the change in average 24-hour SAP (A), DAP (B), HR (C), and activity (D) from baseline at days 3, 5, 7, 12, 15, 19, 22, 25 and 28 in vehicle-treated normotensive mice (n=8, blue unfilled circles), Cmpd17b-treated normotensive mice (n=6, blue filled triangles), vehicle-treated hypertensive mice (n=6, red unfilled circles) and Cmpd17b-treated hypertensive mice (n=6, red filled triangles). The dotted line indicates the time treatment commenced. Histograms (right) represent the average change from baseline across the entire 4 week-period. Data presented as mean ± SEM. **P<0.05, ***P<0.001* for between group comparison. Statistical analysis was conducted with a mixed model split plot analysis of variance corrected with Bonferroni and Greenhouse Geisser adjustments. SAP: systolic arterial pressure; DAP: diastolic arterial pressure; HR: heart rate; Ang II: angiotensin II; Veh: vehicle; Cmpd17b: compound 17b, Vehicle-treated normotensive mice: saline-infused vehicle-treated mice, Cmpd17b-treated normotensive mice: saline-infused mice treated with Cmpd17b, Vehicle-treated hypertensive mice: Ang II-infused vehicle-treated mice, Cmpd17b-treated hypertensive mice: Ang II-infused mice treated with Cmpd17b.

### *Supplementary Figure S6 Chronic effect of Cmpd17b or vehicle on cardiovascular parameters and locomotor activity over 28 days in hypertensive and normotensive mice.*

Line graphs (left) show average of 24-hour absolute MAP (A), SAP (B), DAP (C), HR (D), and activity (E) from baseline at days 3, 5, 7, 12, 15, 19, 22, 25 and 28 in vehicle-treated normotensive mice (n=8, blue unfilled circles), Cmpd17b-treated normotensive mice (n=6, blue filled triangles), vehicle-treated hypertensive mice (n=6, red unfilled circles) and Cmpd17b-treated hypertensive mice (n=6, red filled triangles). The dotted line indicates the time treatment commenced. Histograms (right) represent the average change from baseline across the entire 4 week-period. Data presented as mean ± SEM. **P<0.05, ***P<0.001* for between group comparison. Statistical analysis was conducted with a mixed model split plot analysis of variance corrected with Bonferroni and Greenhouse Geisser adjustments. MAP: mean arterial pressure; SAP: systolic arterial pressure; DAP: diastolic arterial pressure; HR: heart rate; Ang II: angiotensin II; Veh: vehicle; Cmpd17b: compound 17b, Vehicle-treated normotensive mice: saline-infused vehicle-treated mice, Cmpd17b-treated normotensive mice: saline-infused mice treated with Cmpd17b, Vehicle-treated hypertensive mice: Ang II-infused vehicle-treated mice, Cmpd17b-treated hypertensive mice: Ang II-infused mice treated with Cmpd17b.

### *Supplementary Figure S7 Acute effect of losartan and Ang II on MAP in Ang II-induced hypertensive and normotensive mice treated with vehicle or Cmpd17b.*

Line graphs show (A) the 10-min average MAP over 60 mins before and after injection of losartan (10mg/kg, i.p.; dotted line) and (B) the 30-second average MAP for 3 mins before and after injection of Ang II (0.2mg/kg, i.p. dotted line) in vehicle-treated normotensive mice (n=6, blue unfilled circles), Cmpd17b-treated normotensive mice (n=7, blue filled triangles), vehicle-treated hypertensive mice (n=4, red unfilled circles) and Cmpd17b-treated hypertensive mice (n=6, red filled triangles). Hatched panels (A) indicate the data periods analysed. Top histograms represent the average change in MAP 40~60 minutes post injection of losartan compared to 60-minute before injection), and bottom histograms represent the average change in MAP 3 minutes before and after injection of Ang II. Data presented as mean ± SEM. ****P<0.001 for between group comparison.* Statistical analysis was conducted with mixed model split plot analysis of variance corrected with Bonferroni and Greenhouse Geisser adjustments. MAP: mean arterial pressure; Cmpd17b: compound 17b; Ang II: angiotensin II; i.p.: intraperitoneal; Vehicle-treated normotensive mice: saline-infused vehicle-treated mice, Cmpd17b-treated normotensive mice: saline-infused mice treated with Cmpd17b, Vehicle-treated hypertensive mice: Ang II-infused vehicle-treated mice, Cmpd17b-treated hypertensive mice: Ang II-infused mice treated with Cmpd17b.

**(A)**

**(B)**

### *Supplementary Figure S8 Chronic Cmpd17b treatment blunts renal mass but not glomerulosclerosis index in Ang II-induced hypertensive mice.*

Kidney weight normalised to body weight (mg/g) was recorded (A). The glomerulosclerosis index of the kidney was calculated using PAS stain (B). Histograms (A-B) represent vehicle-treated normotensive mice (n=8-11, blue unfilled), Cmpd17b-treated normotensive mice (n=8-12, blue filled), vehicle-treated hypertensive mice (n=9-12, red unfilled) and Ang II-infused Cmpd17b-treated mice (n=9-12, red filled). The scale bar in black represents 1mm. Results presented as mean ± SEM. Statistical analysis was performed using a two-way ANOVA followed by Bonferroni significant difference post-hoc test*. *P<0.05, **P<0.01* for differences between groups. Ang II: angiotensin II, Veh: vehicle, GSI: glomerulosclerosis index, Cmpd17b: compound 17b, PAS: periodic acid-schiff, saline: normotensive mice, Ang II: hypertensive mice.

**
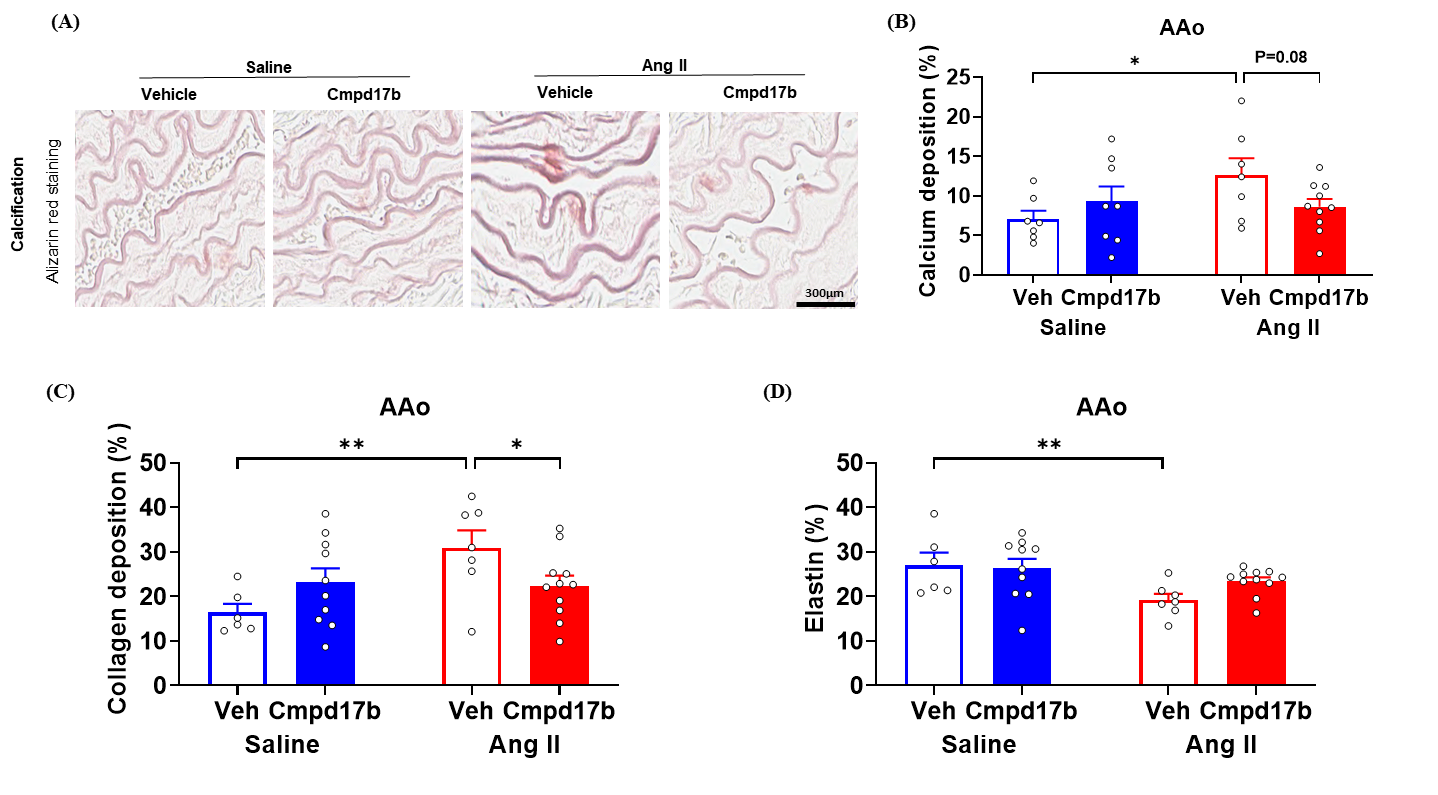
**

### *Supplementary Figure S9 Chronic Cmpd17b treatment blunts collagen and calcium deposition but not elastin percentage in Ang II-induced hypertensive mice.*

Quantification of calcium was performed by Alizarin Red stain. Representative images of abdominal aorta show red-stained calcification (A-B). Quantification of the collagen (fibrotic) area and elastin percentage of the abdominal aorta was performed by pentachrome stain (C-D). Histograms (B-D) represent vehicle-treated normotensive mice (n=6-7, blue unfilled), Cmpd17b-treated normotensive mice (n=8-10, blue filled), vehicle-treated hypertensive mice (n=7, red unfilled) and Ang II-infused Cmpd17b-treated mice (n=10-11, red filled). The scale bar in black represents 300 µm. Results presented as mean ± SEM. Statistical analysis was performed using a two-way ANOVA followed by Bonferroni significant difference post-hoc test*. *P<0.05, **P<0.01* for differences between groups. Ang II: angiotensin II, Veh: vehicle, Cmpd17b: compound 17b, saline: normotensive mice, Ang II: hypertensive mice, AAo: abdominal aorta.

negative_log10_of_adjusted_p_value


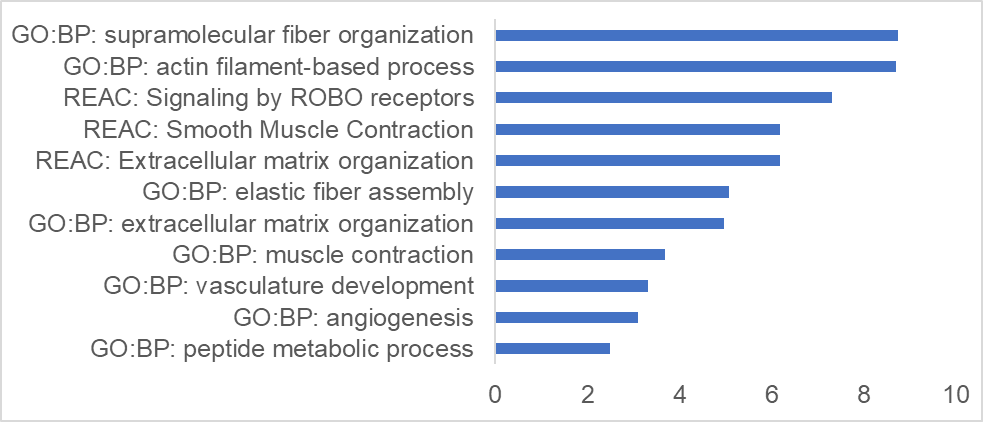


negative_log10_of_adjusted_p_value


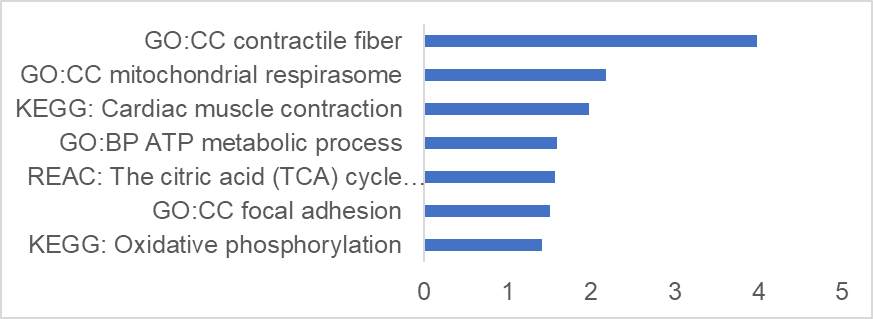


### *Supplementary Figure S10 Commonly identified enrichment pathways in human hypertension and Cmpd17b-treated hypertensive mice.*


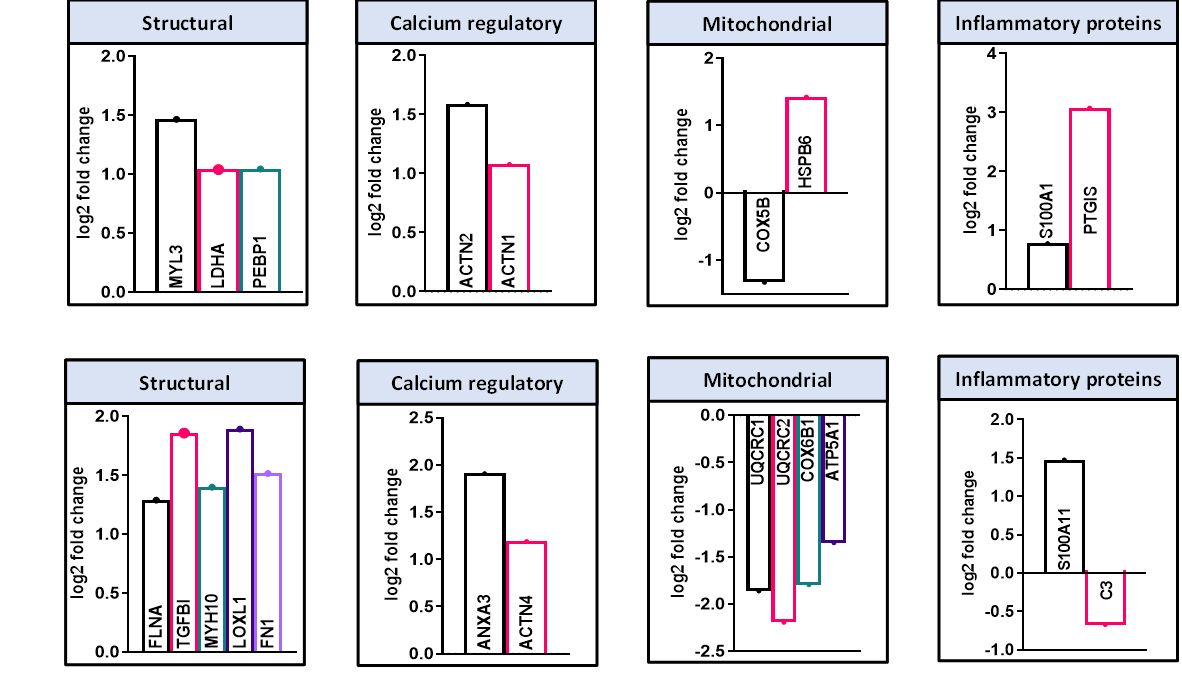


### *Supplementary Figure S11 Significantly changed structural, calcium regulatory and mitochondrial proteins in heart and aorta of human hypertensive datasets.*

Histograms represent the log2fold change of structural, calcium regulatory and mitochondrial proteins in heart (top) and aorta (bottom). All the proteins displayed in the graph were significantly differentiated (*P<0.05*) in human hypertensive heart and aorta compared with controls.


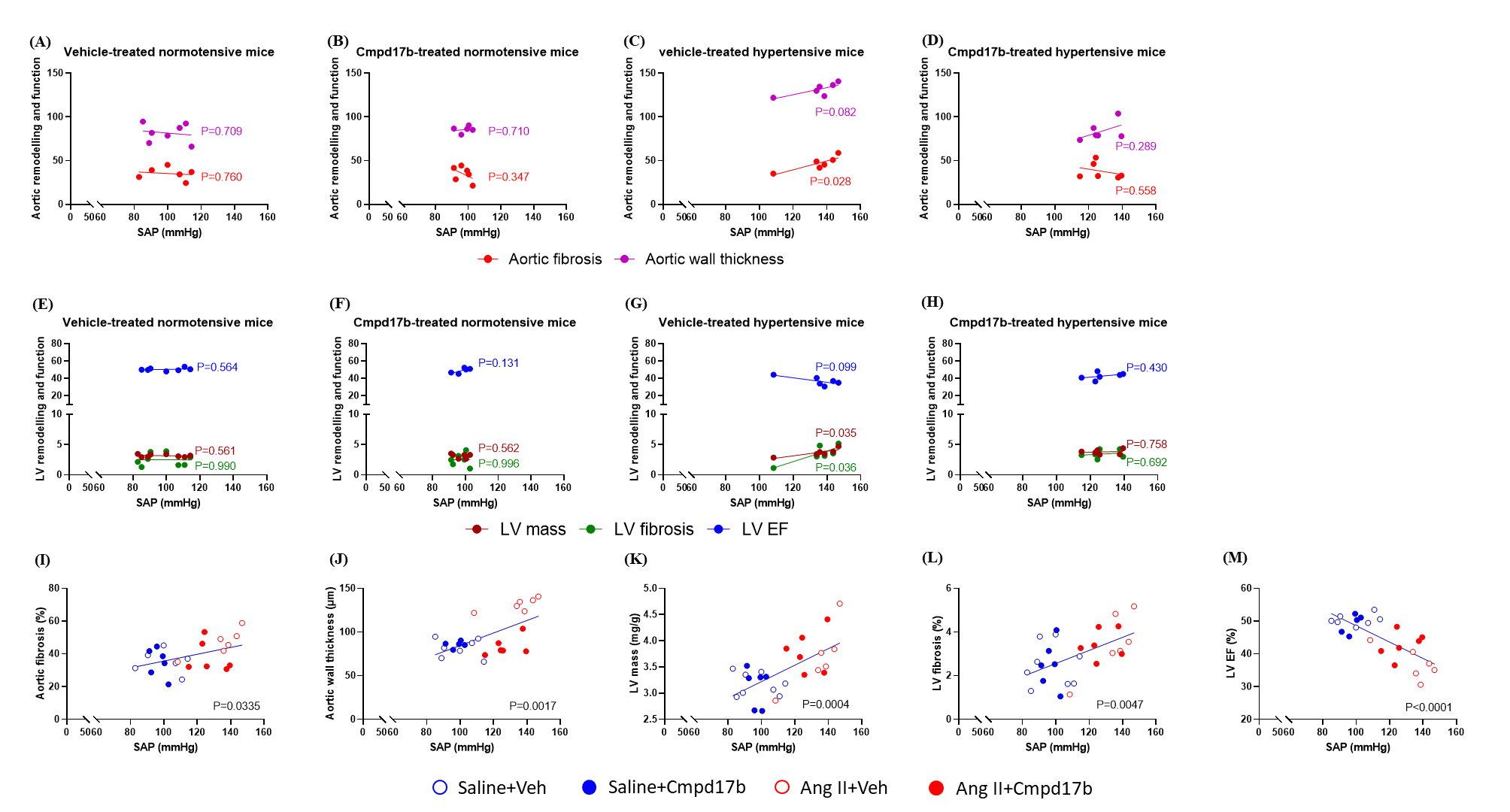


### *Supplementary Figure* *S12* *Correlation of blood pressure with cardiovascular remodelling and function in Ang II-induced hypertensive mice treated with vehicle and Cmpd17b.*

Line graphs represent the correlation of blood pressure with aortic fibrosis (n=6, red circles), and wall thickness (n=5-7, purple circles) and with cardiac mass (n=6-8, maroon circles), fibrosis (n=6-8, green circles), and ejection fraction (n=5-7, blue circles) in normotensive and hypertensive mice treated with vehicle and Cmpd17b (A-H). Line graphs (I-M) represent the correlation between blood pressure and aortic fibrosis and wall thickness and cardiac mass, fibrosis, and ejection fraction in saline infused mice treated with vehicle (n=8, blue unfilled circles) and Cmpd17b (n=6, blue filled circles) and Ang II infused mice treated with vehicle (n=6, red unfilled circles) and Cmpd17b (n=6, red filled circles). Results presented as data of individual animals. Significance was calculated using Pearson r correlation analysis and showed as number for differences between comparison. Normotensive mice: saline infused mice, hypertensive mice: Ang II infused mice, LV: left ventricle, SAP: systolic arterial pressure, EF: ejection fraction, Veh: vehicle, Sal: saline, Ang II: angiotensin II, Cmpd17b: compound 17b.

1. SUPPLEMENTARY TABLES

| **Vehicle-treated normotensive mice: (n=8)** | | | | | | | | | | | |
| --- | --- | --- | --- | --- | --- | --- | --- | --- | --- | --- | --- |
|  | Week 0 | Day 3 | Day 5 | Day 7 | Day 12 | Day 15 | Day 19 | Day 22 | Day 26 | Day 28 | *P_treatment_* |
| MAP (mmHg) | 92 ± 3 | 91 ± 2 | 91 ± 3 | 90 ± 3 | 91 ± 3 | 91 ± 2 | 91 ± 3 | 90 ± 3 | 91 ± 3 | 90 ± 3 | >0.5 |
| HR (b/min) | 483 ± 16 | 498 ± 14 | 498 ± 13 | 503 ± 12 | 496 ± 13 | 502 ± 13 | 494 ± 13 | 502 ± 15 | 499 ± 16 | 505 ± 16 | **0.02** |
| Activity (units) | 0.8 ± 0.5 | 0.6 ± 0.4 | 0.5 ± 0.4 | 0.6 ± 0.4 | 0.5 ± 0.4 | 0.5 ± 0.4 | 0.5 ± 0.3 | 0.6 ± 0.4 | 0.5 ± 0.4 | 0.5 ± 0.4 | 0.23 |
| BW (g) | 29 ± 1 | 30 ± 1 | 30 ± 1 | 30 ± 1 | 31 ± 1 | 31 ± 1 | 31 ± 1 | 31 ± 1 | 31 ± 1 | 31 ± 1 | **<0.001** |
| **Cmpd17b-treated normotensive mice (n=6)** | | | | | | | | | | | |
|  | Week 0 | Day 3 | Day 5 | Day 7 | Day 12 | Day 15 | Day 19 | Day 22 | Day 26 | Day 28 | *P_treatment_* |
| MAP (mmHg) | 88 ± 2 | 88 ± 2 | 87 ± 2 | 85 ± 2 | 86 ± 2 | 86 ± 2 | 88 ± 2 | 85 ± 2 | 86 ± 2 | 87 ± 2 | >0.5 |
| HR (b/min) | 488 ± 17 | 500 ± 11 | 509 ± 11 | 507 ± 13 | 499 ± 13 | 496 ± 11 | 492 ± 12 | 487 ± 12 | 476 ± 12 | 486 ± 10 | >0.5 |
| Activity (units) | 0.6 ± 0.3 | 1 ± 0.2 | 1 ± 0.2 | 0.9 ± 0.1 | 0.8 ± 0.2 | 0.8 ± 0.1 | 0.9 ± 0.1 | 0.9 ± 0.2 | 0.7 ± 0.1 | 0.8 ± 0.1 | 0.31 |
| BW (g) | 31 ± 0 | 31 ± 1 | 31 ± 1 | 32 ± 0 | 33 ± 0 | 33 ± 0 | 33 ± 0 | 33 ± 0 | 32 ± 0 | 32 ± 0 | **<0.001** |
| **Vehicle-treated hypertensive mice (n=6)** | | | | | | | | | | | |
|  | Week 0 | Day 3 | Day 5 | Day 7 | Day 12 | Day 15 | Day 19 | Day 22 | Day 26 | Day 28 | *P_treatment_* |
| MAP (mmHg) | 92 ± 3 | 111 ± 4 | 114 ± 4 | 112 ± 4 | 121 ± 4 | 124 ± 3 | 125 ± 3 | 122 ± 4 | 122 ± 4 | 121 ± 4 | **<0.001** |
| HR (b/min) | 497 ± 17 | 512 ± 14 | 523 ± 16 | 526 ± 16 | 498 ± 13 | 511 ± 13 | 509 ± 12 | 497 ± 12 | 503 ± 15 | 495 ± 10 | 0.28 |
| Activity (units) | 0.5 ± 0.2 | 0.9 ± 0.2 | 0.7 ± 0.2 | 0.8 ± 0.2 | 0.6 ± 0.2 | 0.7 ± 0.2 | 0.7 ± 0.2 | 0.8 ± 0.2 | 0.6 ± 0.2 | 0.7 ± 0.2 | >0.5 |
| BW (g) | 29 ± 1 | 29 ± 1 | 29 ± 1 | 29 ± 1 | 30 ± 1 | 30 ± 1 | 30 ± 1 | 30 ± 1 | 30 ± 1 | 30 ± 1 | **0.028** |
| **Cmpd17b-treated hypertensive mice (n=6)** | | | | | | | | | | | |
|  | Week 0 | Day 3 | Day 5 | Day 7 | Day 12 | Day 15 | Day 19 | Day 22 | Day 26 | Day 28 | *P_treatment_* |
| MAP (mmHg) | 92 ± 3 | 115 ± 5 | 109 ± 5 | 108 ± 4 | 115 ± 3 | 118 ± 3 | 115 ± 3 | 115 ± 3 | 118 ± 3 | 114 ± 2 | **<0.001** |
| HR (b/min) | 500 ± 13 | 510 ± 12 | 501 ± 14 | 502 ± 14 | 506 ± 10 | 514 ± 8 | 511 ± 7 | 478 ± 11 | 499 ± 10 | 499 ± 10 | >0.5 |
| Activity (units) | 0.6 ± 0.3 | 0.7 ± 0.5 | 0.6 ± 0.5 | 0.8 ± 0.5 | 0.8 ± 0.5 | 0.8 ± 0.5 | 0.8 ± 0.5 | 1.2 ± 0.6 | 0.8 ± 0.5 | 0.8 ± 0.5 | >0.5 |
| BW (g) | 31 ± 0 | 31 ± 1 | 31 ± 1 | 31 ± 1 | 32 ± 1 | 32 ± 1 | 32 ± 1 | 33 ± 0 | 31 ± 0 | 31 ± 0 | **0.011** |

### *Supplementary Table S1 Effect of Cmpd17b or vehicle on cardiovascular parameters in hypertensive and normotensive mice.*

Results presented as Mean ± SEM. Statistical analysis conducted by mixed model split plot analysis of variance corrected with Bonferroni and Greenhouse Geisser adjustments. P_treatment_ is change from baseline over 4-week period. Exact probabilities shown when P=0.001-0.5. Probabilities are in bold when P<0.05 and considered to be statistically significant. MAP: mean arterial pressure; HR: heart rate; BW: body weight, Vehicle-treated normotensive mice: saline-infused vehicle-treated mice, Cmpd17b-treated normotensive mice: saline-infused mice treated with Cmpd17b, Vehicle-treated hypertensive mice: Ang II-infused vehicle-treated mice, Cmpd17b-treated hypertensive mice: Ang II-infused mice treated with Cmpd17b.

### *Supplementary Table S2 Common and significant proteins in LV and TA of mouse and human hypertension.*

All the proteins displayed in the table were significantly differentiated (P<0.05) in human hypertensive heart and aorta compared with controls and similar proteins were identified in Ang II induced hypertensive mice.

| **LV proteins in hypertrophic heart** | **TA proteins in aortic remodelling** | | |
| --- | --- | --- | --- |
| Acadm | Acadm | Erap1 | Pdlim5 |
| Actn2 | Acat1 | Fah | Pfkl |
| Aifm1 | Acat2 | Fasn | Pfkp |
| Aldh2 | Acly | Fbln2 | Pgd |
| Anxa6 | Aco1 | Fermt3 | Pls3 |
| Csrp3 | Actn4 | Flnc | Ppib |
| Etfa | Agl | Fn1 | Ppp1r12a |
| Fhl1 | Aldoa | Galm | Prkaca |
| Fhl2 | Anxa1 | Glod4 | Psmd3 |
| Flnc | Arhgap1 | Gpd1 | Pygl |
| Gstm2 | Cap1 | Gpi | Rnpep |
| Gsto1 | Cast | Hibch | Rrbp1 |
| Hspb7 | Cfl2 | Hnrnpul2 | Samhd1 |
| Ldb3 | Ckap4 | Hsd17b12 | Sec22b |
| Ldha | Cnn1 | Hspa1a | Sec23a |
| Map4 | Cnn2 | Hsph1 | Septin8 |
| Myh7 | Col15a1 | Htra1 | Serpinh1 |
| Myom1 | Col18a1 | Igfbp7 | Srsf1 |
| Pcbp1 | Cops2 | Lonp1 | Ssb |
| Pgm5 | Cops4 | Lrp1 | Suclg1 |
| Tgm2 | Dhrs7 | Opa1 | Tagln |
| Vdac3 | Eif4h | Palld | Tkt |
| Wdr1 | Eno1 | Pdia3 | Twf2 |
| Xirp1 | Ephx1 | Pdlim1 | Ugp2 |

### *Supplementary Table S3 Full name of all the listed proteins in proteome analysis.*

| Acacb | Acetyl-CoA carboxylase 2 |
| --- | --- |
| Acadm | Medium-chain specific acyl-CoA dehydrogenase, mitochondrial |
| Acat1 | Acetyl-CoA acetyltransferase, mitochondrial |
| Acat2 | Acetyl-CoA acetyltransferase, cytosolic |
| Acsf3 | Malonate--CoA ligase ACSF3, mitochondrial |
| Acta1 | Actin, alpha skeletal muscle |
| Acta1 | Actin, alpha skeletal muscle |
| Actn2 | Alpha-actinin-2 |
| Actn4 | Alpha-actinin-4 |
| Agpat1 | 1-acyl-sn-glycerol-3-phosphate acyltransferase alpha |
| Aifm1 | Apoptosis-inducing factor 1, mitochondrial |
| Aldh2 | Aldehyde dehydrogenase, mitochondrial |
| Anxa1 | Annexin A1 |
| Anxa11 | Annexin A11 |
| Anxa3 | Annexin A3 |
| Anxa4 | Annexin A4 |
| Anxa5 | Annexin A5 |
| Anxa6 | Annexin A6 |
| Apool | MICOS complex subunit Mic27 |
| Arf1 | ADP-ribosylation factor 1 |
| Bdh1 | D-beta-hydroxybutyrate dehydrogenase, mitochondrial |
| C1qbp | Complement component 1 Q subcomponent-binding protein, mitochondrial |
| C3 | Complement C3 |
| Cav3 | Caveolin-3 |
| Cd47 | Leukocyte surface antigen CD47 |
| Cdh2 | Cadherin-2 |
| Cfh | Complement factor H |
| Cfl2 | Cofilin-2 |
| Ckap4 | Cytoskeleton-associated protein 4 |
| Clpp | ATP-dependent Clp protease proteolytic subunit, mitochondrial |
| Col15a1 | Collagen alpha-1 (XV) chain |
| Col18a1 | Collagen alpha-1 (XVIII) chain |
| Coq8a | Atypical kinase COQ8A, mitochondrial |
| Cox5b | Cytochrome c oxidase subunit 5B, mitochondrial |
| Crat | Carnitine O-acetyltransferase |
| Cyb5r1 | NADH-cytochrome b5 reductase 1 |
| Dap3 | 28S ribosomal protein S29, mitochondrial |
| Dysf | Dysferlin |
| Egfr | Epidermal growth factor receptor |
| Endog | Endonuclease G, mitochondrial |
| Etfa | Electron transfer flavoprotein subunit alpha, mitochondrial |
| Fahd1 | Acylpyruvase FAHD1, mitochondrial |
| Fasn | Fatty acid synthase |
| Fbln2 | Fibulin-2 |
| Fech | Ferrochelatase, mitochondrial |
| Fhl1 | Four and a half LIM domains protein 1 |
| Fhl2 | Four and a half LIM domains protein 2 |
| Fkbp1a | Peptidyl-prolyl cis-trans isomerase FKBP1A |
| Fkbp4 | Peptidyl-prolyl cis-trans isomerase FKBP4 |
| Flna | Filamin-A |
| Flnc | Filamin-C |
| Fn1 | Fibronectin |
| Gfm2 | Ribosome-releasing factor 2, mitochondrial |
| Gpi | Glucose-6-phosphate isomerase |
| Gpx1 | Glutathione peroxidase 1 |
| Gstm1 | Glutathione S-transferase Mu 1 |
| Gstm2 | Glutathione S-transferase Mu 2 |
| Hibch | 3-hydroxyisobutyryl-CoA hydrolase, mitochondrial |
| Hint2 | Histidine triad nucleotide-binding protein 2, mitochondrial |
| Hspa1a | Heat shock 70 kDa protein 1A |
| Hspa4 | Heat shock 70 kDa protein 4 |
| Hspa9 | Stress-70 protein, mitochondrial |
| Hspb6 | Heat shock protein beta-6 |
| Hspb7 | Heat shock protein beta-7 |
| Itih4 | Inter alpha-trypsin inhibitor, heavy chain 4 |
| Kng1 | Kininogen-1 |
| L2hgdh | L-2-hydroxyglutarate dehydrogenase, mitochondrial |
| Ldb3 | LIM domain-binding protein 3 |
| Ldha | L-lactate dehydrogenase A chain |
| Letm1 | Mitochondrial proton/calcium exchanger protein |
| LMO7 | LIM (Lin11, Isl-1 & Mec-3) domain protein 7 |
| Loxl1 | Lysyl oxidase homolog 1 |
| Lrp1 | Prolow-density lipoprotein receptor-related protein 1 |
| Ltbp4 | Latent-transforming growth factor beta-binding protein 4 |
| Map4 | Microtubule-associated protein |
| Mccc2 | Methylcrotonoyl-CoA carboxylase beta chain, mitochondrial |
| Mdh2 | Malate dehydrogenase, mitochondrial |
| Mecr | Enoyl-[acyl-carrier-protein] reductase, mitochondrial |
| Memo1 | Mediator of cell motility 1 |
| Mff | Mitochondrial fission factor |
| Mif | Macrophage migration inhibitory factor |
| Mrpl4 | 39S ribosomal protein L4, mitochondrial |
| Mrpl45 | 39S ribosomal protein L45, mitochondrial |
| Mrps27 | 28S ribosomal protein S27, mitochondrial |
| Msra | Mitochondrial peptide methionine sulfoxide reductase |
| Msrb2 | Methionine-R-sulfoxide reductase B2, mitochondrial |
| Mtarc1 | Mitochondrial amidoxime-reducing component 1 |
| Myh10 | Myosin-10 |
| Myh11 | Myosin-11 |
| Myh7 | Myosin-7 |
| Mylk3 | Myosin light chain kinase 3 |
| Myo18a | Unconventional myosin-XVIIIa |
| Myom1 | Myomesin 1 |
| Naa15 | N-alpha-acetyltransferase 15, NatA auxiliary subunit |
| Ndufa13 | NADH dehydrogenase [ubiquinone] 1 alpha subcomplex subunit 13 |
| Ndufa7 | NADH dehydrogenase [ubiquinone] 1 alpha subcomplex subunit 7 |
| Ndufb11 | NADH dehydrogenase [ubiquinone] 1 beta subcomplex subunit 11 mitochondrial |
| Ndufb4 | NADH dehydrogenase [ubiquinone] 1 beta subcomplex subunit 4 |
| Ndufb6 | NADH dehydrogenase [ubiquinone] 1 beta subcomplex subunit 6 |
| Ndufb9 | NADH dehydrogenase [ubiquinone] 1 beta subcomplex subunit 9 |
| Ndufs2 | NADH dehydrogenase [ubiquinone] iron-sulfur protein 2, mitochondrial |
| Ndufs3 | NADH dehydrogenase [ubiquinone] iron-sulfur protein 3, mitochondrial |
| Ndufs7 | NADH dehydrogenase [ubiquinone] iron-sulfur protein 7, mitochondrial |
| Ndufv1 | NADH dehydrogenase [ubiquinone] flavoprotein 1, mitochondrial |
| Ndufv2 | NADH dehydrogenase [ubiquinone] flavoprotein 2, mitochondrial |
| Nfs1 | Cysteine desulfurase, mitochondrial |
| Nln | Neurolysin, mitochondrial |
| Nlrx1 | NLR family member X1 |
| Nrap | Nebulin-related-anchoring protein |
| Opa1 | Dynamin-like 120 kDa protein, mitochondrial |
| Pcbp1 | Poly(rC)-binding protein 1 |
| Pcca | Propionyl-CoA carboxylase alpha chain, mitochondrial |
| Pccb | Propionyl-CoA carboxylase beta chain, mitochondrial |
| Pebp1 | Phosphatidylethanolamine-binding protein 1 |
| Pfkp | ATP-dependent 6-phosphofructokinase, platelet type |
| Pgd | 6-phosphogluconate dehydrogenase, decarboxylating |
| Plaa | Phospholipase A-2-activating protein |
| Pls3 | Plastin-3 |
| Ppp1r12a | Protein phosphatase 1 regulatory subunit 12A |
| Prdx6 | Peroxiredoxin-6 |
| Ptges2 | Prostaglandin E synthase 2 |
| Ptges3 | Prostaglandin E synthase 3 |
| Ptgis | Prostacyclin synthase |
| Ptgr2 | Prostaglandin reductase 2 |
| Rdh13 | Retinol dehydrogenase 13 |
| Serpina1b | Alpha-1-antitrypsin 1-2 |
| Serpinc1 | Antithrombin-III |
| Serpinh1 | Serpin H1 |
| Sirt5 | NAD-dependent protein deacylase sirtuin-5, mitochondrial |
| Slc25a1 | Tricarboxylate transport protein, mitochondrial |
| Slc8a1 | Sodium/calcium exchanger 1 |
| Slc9a3r2 | Na(+)/H(+) exchange regulatory cofactor NHE-RF2 |
| Snta1 | Alpha-1-syntrophin |
| Sod1 | Superoxide dismutase [Cu-Zn] |
| Sod2 | Superoxide dismutase [Mn], mitochondrial |
| Sorbs2 | Sorbin And SH3 Domain Containing 2 |
| Sucla2 | Succinate--CoA ligase [ADP-forming] subunit beta, mitochondrial |
| Suclg1 | Succinate--CoA ligase [ADP/GDP-forming] subunit alpha, mitochondrial |
| Suclg2 | Succinate--CoA ligase [GDP-forming] subunit beta, mitochondrial |
| Synpo | Synaptopodin |
| Synpo2l | Synaptopodin 2-like protein |
| Tagln | Transgelin |
| Tagln2 | Transgelin-2 |
| Taldo1 | Transaldolase |
| Tgfb1i1 | Transforming growth factor beta-1-induced transcript 1 protein |
| Tgfbi | Transforming growth factor-beta-induced protein ig-h3 |
| Tgm2 | Protein-glutamine gamma-glutamyltransferase 2 |
| Timm17a | Mitochondrial import inner membrane translocase subunit Tim17-A |
| Tnnc1 | Troponin C, slow skeletal and cardiac muscles |
| Tomm20 | Mitochondrial import receptor subunit TOM20 homolog |
| Ttn | Titin |
| Ucp1 | Mitochondrial brown fat uncoupling protein 1 |
| Vcl | Vinculin |
| Vdac3 | Voltage-dependent anion-selective channel protein 3 |
| Wdr1 | WD repeat-containing protein 1 |
| Xirp1 | Xin actin-binding repeat-containing protein 1 |
| Xirp2 | Xin actin-binding repeat-containing protein 2 |

| **Parameter** | **Normotensive mice** | | **Hypertensive mice** | |
| --- | --- | --- | --- | --- |
|  | **Vehicle** | **Cmpd17b** | **Vehicle** | **Cmpd17b** |
| Heart Rate (bpm) | 453 ± 9 | 488 ± 11† | 550 ± 10*** | 554 ± 11### |
| Area,_s_ (mm^2^) | 16.3 ± 0.4 | 16.7 ± 0.3 | 19.5 ± 0.5*** | 18.0 ± 0.6$ |
| Area,_d_ (mm^2^) | 24.5 ± 0.5 | 25.0 ± 0.4 | 25.6 ± 0.6 | 25.4 ± 0.5 |
| Volume,_s_ (µL) | 32.5 ± 1.0 | 34.0 ± 1.0 | 43.7 ± 1.7*** | 37.5 ± 1.6$$ |
| Volume,_d_ (µL) | 65.1 ± 1.7 | 67.2 ± 1.2 | 69.4 ± 2.0 | 67.7 ± 1.7 |
| Stroke volume (µL) | 32.6 ± 0.8 | 33.3 ± 0.7 | 25.7 ± 1.0*** | 30.2 ± 0.9$$$# |
| Ejection fraction (%) | 50.1 ± 0.6 | 49.5 ± 1.0 | 37.1 ± 1.3*** | 44.7 ± 1.4$$$## |
| Cardiac output (mL/min) | 14.8 ± 0.4 | 16.3 ± 0.6 | 14.1 ± 0.5 | 16.7 ± 0.5$$$ |
| Area change (%) | 33.5 ± 0.4 | 32.9 ± 0.9 | 23.8 ± 1.0*** | 30.5 ± 2.2$$ |

### *Supplementary Table S4 B-mode echocardiography analysis of LV in hypertensive and normotensive mice treated with vehicle or Cmpd17b.*

Results are presented as mean ± SEM. Statistical analysis was performed using a two-way ANOVA followed by Bonferroni significant difference post-hoc test. ****P<0.001 for differences between normotensive and hypertensive mice treated with vehicle.* †P<0.05 *for differences between normotensive mice treated with vehicle and Cmpd17b. #P<0.05, ##P<0.01, ###P<0.001 for differences between normotensive and hypertensive mice treated with Cmpd17b. $P<0.05, $$P<0.01, $$$P<0.001 for differences between hypertensive mice treated with vehicle and Cmpd17b.* LV: left ventricle, s: systolic, d: diastolic, bpm: beats per minute, Cmpd17b: compound 17b, vehicle-treated normotensive mice (n=9): saline-infused vehicle-treated mice, Cmpd17b-treated normotensive mice (n=10): saline-infused mice treated with Cmpd17b, vehicle-treated hypertensive mice (n=10): Ang II-infused vehicle-treated mice, Cmpd17b-treated hypertensive mice (n=11): Ang II-infused mice treated with Cmpd17b.

| **Parameter** | **Normotensive mice** | | **Hypertensive mice** | |
| --- | --- | --- | --- | --- |
|  | **Vehicle** | **Cmpd17b** | **Vehicle** | **Cmpd17b** |
| IVS_d_ (mm) | 0.91 ± 0.03 | 0.94 ± 0.03 | 1.25 ± 0.02*** | 1.14 ± 0.04$### |
| LVID_d_ (mm) | 3.8 ± 0.1 | 3.8 ± 0.1 | 3.2 ± 0.1** | 3.5 ± 0.2 |
| LVID_s_ (mm) | 2.5 ± 0.1 | 2.5 ± 0.1 | 2.4 ± 0.1 | 2.4 ± 0.2 |
| LVPW_d_ (mm) | 0.91 ± 0.03 | 0.94 ± 0.03 | 1.26 ± 0.02*** | 1.13 ± 0.03$$### |
| FS (%) | 33.9 ± 1.1 | 35.0 ± 1.8 | 24.3 ± 1.2*** | 32.0 ± 2.2$$ |
| LV mass (mg) | 131 ± 9 | 135 ± 7 | 165 ± 9** | 159 ± 8# |
| TWT (mm) | 0.91 ± 0.03 | 0.94 ± 0.03 | 1.26 ± 0.02*** | 1.14 ± 0.03$$### |
| LVRI (mg/mm) | 34.1 ± 1.8 | 35.7 ± 1.4 | 51 ± 1.4*** | 45.3 ± 1.8$## |
| LV h/r (ratio) | 0.48 ± 0.03 | 0.51 ± 0.03 | 0.79 ± 0.04*** | 0.66 ± 0.04$## |

### *Supplementary Table S5 M-mode echocardiography analysis of LV in hypertensive and normotensive mice treated with vehicle or Cmpd17b.*

Results are presented as mean ± SEM. Statistical analysis was performed using a two-way ANOVA followed by Bonferroni significant difference post-hoc test. ***P<0.01, ***P<0.001 for differences between normotensive and hypertensive mice treated with vehicle. ##P<0.01, ###P<0.001 for differences between normotensive and hypertensive mice treated with Cmpd17b. $P<0.05, $$P<0.01 for differences between hypertensive mice treated with vehicle and Cmpd17b.* s: systolic, d: diastolic, Cmpd17b: compound 17b, LV: left ventricle, PW: posterior wall, FS: fractional shortening, ID: internal diameter, TWT: total wall thickness (average of IVS_d_+ LVPW_d_), LVRI: left ventricle remodelling index (LV mass/LVID_d_), LV h/r ratio: LV wall thickness (h)/internal LV chamber radius (R) where radius is LVID_d_ divided by two, vehicle-treated normotensive mice (n=9): saline-infused vehicle-treated mice, Cmpd17b-treated normotensive mice (n=10): saline-infused mice treated with Cmpd17b, vehicle-treated hypertensive mice (n=10): Ang II-infused vehicle-treated mice, Cmpd17b-treated hypertensive mice (n=11): Ang II-infused mice treated with Cmpd17b.

| **Treatment** | **Normotensive mice** | | **Hypertensive mice** | |
| --- | --- | --- | --- | --- |
|  | **Vehicle** | **Cmpd17b** | **Vehicle** | **Cmpd17b** |
| Body Weight (g) | 29.4 ± 0.9 | 30.3 ± 0.6 | 28.2 ± 0.5 | 29.1 ± 0.6 |
| Heart/BW (mg/g) | 4.14 ± 0.1 | 4.28 ± 0.1 | 5.2 ± 0.12*** | 4.71 ± 0.1$$## |
| LV/BW (mg/g) | 3.08 ± 0.08 | 3.09 ± 0.12 | 4.13 ± 0.12*** | 3.64 ± 0.13$$ |
| RV/BW (mg/g) | 0.61 ± 0.04 | 0.82 ± 0.25 | 0.92 ± 0.30 | 0.86 ± 0.28 |
| Atrium/BW (mg/g) | 0.26 ± 0.03 | 0.27 ± 0.02 | 0.28 ± 0.01 | 0.29 ± 0.02 |
| Kidney (mg/g) | 6.19 ± 0.13 | 6.18 ± 0.19 | 5.38 ± 0.17** | 6.01 ± 0.14$$ |
| Lungs/BW (mg/g) | 5.31 ± 0.09 | 5.18 ± 0.08 | 5.78 ± 0.18* | 5.66 ± 0.14## |
| Liver/BW (mg/g) | 38 ± 2 | 39 ± 2 | 38 ± 2 | 37 ± 2 |
| Spleen/BW (mg/g) | 2.8 ± 0.1 | 3.2 ± 0.1† | 2.8 ± 0.1 | 3.2 ± 0.2$ |

### *Supplementary Table S6 Body weight-normalised organ weights in hypertensive and normotensive mice treated with Cmpd17b or vehicle.*

Results are presented as mean ± SEM. Statistical analysis was performed using a two-way ANOVA followed by Bonferroni significant difference post-hoc test. **P<0.05, **P<0.01, ***P<0.001 for differences between normotensive and hypertensive mice treated with vehicle.* †P<0.05 *for differences between normotensive mice treated with vehicle and Cmpd17b. ##P<0.01 for differences between normotensive and hypertensive mice treated with Cmpd17b. $P<0.05, $$P<0.01 for differences between hypertensive mice treated with vehicle and Cmpd17b.* BW: bodyweight; LV: left ventricle; RV: right ventricle; Cmpd17b: compound 17b, vehicle-treated normotensive mice (n=11): saline-infused vehicle-treated mice, Cmpd17b-treated normotensive mice (n=12): saline-infused mice treated with Cmpd17b, vehicle-treated hypertensive mice (n=12): Ang II-infused vehicle-treated mice, Cmpd17b-treated hypertensive mice (n=12): Ang II-infused mice treated with Cmpd17b.

|  |  | **Normotensive mice** | | **Hypertensive mice** | |
| --- | --- | --- | --- | --- | --- |
| **Parameter** | | **Vehicle** | **Cmpd17b** | **Vehicle** | **Cmpd17b** |
| Strain (%) | | 34 ± 1 | 35 ± 2 | 19 ± 2*** | 30 ± 2$$$# |
| Distensibility (1/Mpa) | | 147 ± 7 | 133 ± 5 | 64 ± 5*** | 88 ± 4$$### |
| IMT (mm) | | 0.08 ± 0.003 | 0.08 ± 0.003 | 0.12 ± 0.005*** | 0.09 ± 0.003$$$ |
| Wall thickness (mm) | | 0.15 ± 0.01 | 0.14 ± 0.01 | 0.18 ± 0.01*** | 0.14 ± 0.01$$$ |
| Diameter (mm) | | 0.47 ± 0.01 | 0.46 ± 0.01 | 0.50 ± 0.02 | 0.48 ± 0.01 |
| Area (mm^2^) | | 0.18 ± 0.01 | 0.17 ± 0.01 | 0.21 ± 0.02 | 0.18 ± 0.01 |

### *Supplementary Table S7 Carotid artery characteristics assessed from ultrasound imaging in hypertensive and normotensive mice treated with Cmpd17b or vehicle.*

Results are presented as mean ± SEM. Statistical analysis was performed using a two-way ANOVA followed by Bonferroni significant difference post-hoc test. ****P<0.001 for differences between normotensive and hypertensive mice treated with vehicle. #P<0.05, ###P<0.001 for differences between normotensive and hypertensive mice treated with Cmpd17b. $$P<0.01, $$$P<0.001 for differences between hypertensive mice treated with vehicle and Cmpd17b.* Cmpd17b: compound 17b, IMT: intima medial thickness, vehicle-treated normotensive mice (n=9): saline-infused vehicle-treated mice, Cmpd17b-treated normotensive mice (n=10): saline-infused mice treated with Cmpd17b, vehicle-treated hypertensive mice (n=10): Ang II-infused vehicle-treated mice, Cmpd17b-treated hypertensive mice (n=10): Ang II-infused mice treated with Cmpd17b.

### *Supplementary Table S8 Common and significant proteins in LV and aorta of mouse hypertensive treated with Cmpd17b and human hypertensive datasets.*

All the proteins displayed in the table were significantly differentiated (P<0.05) in human hypertensive heart and aorta compared with controls and similar proteins were identified in Ang II induced hypertensive mice treated with Cmpd17b.

| **Proteome comparison between human hypertension and Cmpd17b-treated mouse hypertension** | | | | | |
| --- | --- | --- | --- | --- | --- |
| **LV proteins** | **TA proteins** | | | | |
| ACTN1 | ACP1 | COL15A1 | LMAN1 | POSTN | SMTN |
| ANXA5 | ACTN4 | COL18A1 | LMNA | PPP1R12A | SOD3 |
| ARF1 | AEBP1 | COPS2 | LMOD1 | PPP1R12B | SORBS3 |
| COX5B | AHNAK | CYB5R3 | LOXL1 | PRKAR2A | SPR |
| HINT2 | AKR7A2 | DPYSL3 | LPP | PTGIS | SPTAN1 |
| HSPB6 | ANK1 | DSTN | LRP1 | PURA | SYNPO2 |
| LDHA | ANP32A | EFEMP1 | LTBP1 | QDPR | TAGLN |
| MYL3 | ANXA6 | EML2 | LTBP4 | RAB14 | TAGLN2 |
| PEBP1 | AOC3 | ENAH | MCAM | RAB7A | TGFB1I1 |
| PGK1 | ASPH | ESYT2 | MFAP4 | RPL18 | TINAGL1 |
| PRDX6 | ATL3 | FAM120A | MFGE8 | RPL3 | TNS1 |
| TNNC1 | ATP6V1B2 | FBLIM1 | MPST | RPL4 | TNXB |
|  | BCAM | FBLN5 | MYH11 | RPL7 | UBE2L3 |
|  | BGN | FERMT2 | MYL6 | RPS18 | VCAN |
|  | C4B | FLNA | MYLK | RPS2 | VCL |
|  | CALD1 | GLO1 | NPEPPS | RPS3A | VCP |
|  | CAPN2 | HP | NPNT | RPS4X | ZYX |
|  | CAPNS1 | HP1BP3 | PA2G4 | RPS5 |  |
|  | CAST | HSD17B4 | PARVA | RPS9 |  |
|  | CBR3 | HSPA1A | PDLIM1 | RRAS |  |
|  | CCT4 | HSPA8 | PDLIM3 | RRAS2 |  |
|  | CLIC4 | HSPB1 | PDLIM7 | S100A1 |  |
|  | CLU | HSPG2 | PGM5 | SERPINC1 |  |
|  | CNN1 | ITGB1 | PLCD1 | SERPINH1 |  |
|  | CNN3 | ITIH4 | PLS3 | SF1 |  |

| **Parameter** | **Normotensive mice** | | **Hypertensive mice** | |
| --- | --- | --- | --- | --- |
|  | **Vehicle** | **Cmpd17b** | **Vehicle** | **Cmpd17b** |
| WBC (K/µL) | 5.8 ± 0.8 | 4.8 ± 0.4 | 3.9 ± 0.7* | 3.8 ± 0.4# |
| Lymphocytes (K/µL) | 4.9 ± 0.8 | 3.6 ± 0.3 | 3.1 ± 0.7* | 2.7 ± 0.3## |
| Monocytes (K/µL) | 0.2 ± 0.1 | 0.3 ± 0.1 | 0.3 ± 0.2 | 0.3 ± 0.1 |
| Neutrophils (K/µL) | 0.3 ± 0.0 | 0.3 ± 0.1 | 0.2 ± 0.1 | 0.2 ± 0.0 |
| Eosinophils (K/µL) | 0.00 ± 0.00 | 0.01 ± 0.01 | 0.00 ± 0.00 | 0.00 ± 0.00 |
| Basophils (K/µL) | 0.4 ± 0.0 | 0.5 ± 0.1 | 0.3 ± 0.0# | 0.6 ± 0.1$$ |
| Lymphocytes (%) | 83.2 ± 2.6 | 75.8 ± 2† | 78.5 ± 2.6 | 70.3 ± 2.1†### |
| Monocytes (%) | 4.9 ± 1.6 | 6.9 ± 1.2 | 9 ± 3.5 | 9.3 ± 1.4 |
| Neutrophils (%) | 4.4 ± 0.8 | 5.9 ± 1.1 | 5.2 ± 0.8 | 5.2 ± 0.7 |
| Eosinophils (%) | 0.0 ± 0.0 | 0.2 ± 0.2 | 0.0 ± 0.0 | 0.1 ± 0.1 |
| Basophils (%) | 7.6 ± 1.1 | 11.2 ± 1.8 | 7.3 ± 1.3 | 15.1 ± 1.8$$$## |

### *Supplementary Table S9 Haematological differential blood cell counts in vehicle or Cmpd17b-treated hypertensive and normotensive mice.*

Results are presented as mean ± SEM. Haematological data at study end point is expressed as either total count (K/µL) or percent total WBCs. Statistical analysis was performed using a two-way ANOVA followed by Bonferroni significant difference post-hoc test. **P<0.05 for differences between normotensive and hypertensive mice treated with vehicle.* †P<0.05 *for differences between normotensive mice treated with vehicle and Cmpd17b. #P<0.05, ##P<0.01, ###P<0.001 for differences between normotensive and hypertensive mice treated with Cmpd17b. $$P<0.01 for differences between hypertensive mice treated with vehicle and Cmpd17b.* WBC: white blood cells; Ang II: angiotensin II; Cmpd17b: compound 17b, vehicle-treated normotensive mice (n=9): saline-infused vehicle-treated mice, Cmpd17b-treated normotensive mice (n=10): saline-infused mice treated with Cmpd17b, vehicle-treated hypertensive mice (n=10): Ang II-infused vehicle-treated mice, Cmpd17b-treated hypertensive mice (n=11): Ang II-infused mice treated with Cmpd17b.

### *Supplementary Table S10 List of gene primers*

| **Genes** | **Human primer sequences (5’-3’)** | |
| --- | --- | --- |
|  | **Forward primer** | **Reverse primer** |
| *NDUFA7* | CATCATGTCGTCGCAGAAGG | GAGGACAGCTCCCACCTCTT |
| *COQ8A* | CAGGAAGAACACCATCAC | TGAGTCATCAGCAACTTAA |
| *PCOL3* | GACTGGAAGAGCGGAGAGTAC | CCTTGATGGGGTCCAGGTT |
| *TIMP2* | CAGAAAAAGCTGGGTCTTGC | AGTGTCCGGAGGCTGAGAA |
| *ANXA1* | GGCCTTGGAACTGATGAAGA | GTTGTGGATAGCTTCTGGTG |
| *LGR6* | GAGATGGAGGACTCAAAGCCA | AGTCCATTGCAGAGCACGGAG |
| *FPR1* | ACCCAGAGCAAGACCACAGC | TCCATCTTGTCTGCTCCTGGA |
| *FPR2* | GCCTTTTGGCTGGTTCCTGTG | CCAGACTGGATGCAGGACACA |
| *TNFa* | CTCGAACCCCGAGTGACAA | GCTGCCCCTCAGCTTGAG |
| *IL1β* | GCTGAGGAAGATGCTGGTTC | TCCATATCCTGTCCCTGGAG |
| *MCP1* | AGCAGCAAGTGTCCCAAAGA | GGTGTCTGGGGAAAGCTAGG |

### *Supplementary Table S11 TA tissue quantitative proteomics for groups WT-Veh, WT-Cmpd17b, Hypertensive-Veh, and Hypertensive-Cmpd17b.*

### *Supplementary Table S12 LV tissue quantitative proteomics for groups WT-Veh, WT-Cmpd17b, Hypertensive-Veh, and Hypertensive-Cmpd17b.*

### *Supplementary Table S13 T-Test analysis of TA tissue proteomics of WT, Cmpd17b and NTN groups. Red highlights p<0.05*

### *Supplementary Table S14 Functional enrichment of proteins from TA tissue proteomics from HTN_Veh vs WT_Veh group.* Increased (high) enrichment in HTN_Veh group.

### *Supplementary Table S15 T-Test analysis of LV tissue proteomics of WT, Cmpd17b and NTN groups. Red highlights p<0.05*

### *Supplementary Table S16 Functional enrichment of proteins from LV tissue proteomics from HTN_Veh vs WT_Veh group.* Increased (high) enrichment in HTN_Veh group.

### *Supplementary Table S17 Functional enrichment of proteins from TA tissue proteomics from HTN_Cmpd17b vs HTN_Veh group.* Increased (high) enrichment in HTN_Cmpd17b group.

### *Supplementary Table S18 Functional enrichment of proteins from LV tissue proteomics from HTN_Cmpd17b vs HTN_Veh group.* Increased (high) enrichment in HTN_Cmpd17b group.

### *Supplementary Table S19 Proteins identified in LV and TA tissue of hypertensive mice+Veh but not in normotensive mice+Veh.*

### *Supplementary Table S20 Differential proteins of HASM cells proteomics from Ang II_Veh vs control_Veh and Ang II_Cmpd17b vs AngII_Veh group.*

### *Supplementary Table S21 Functional enrichment of proteins from HASM cells proteomics from Ang II_Veh vs control_Veh and Ang II_Cmpd17b vs AngII_Veh group.*

### *Supplementary Table S22 Differential proteins of HCF cells proteomics from Ang II_Veh vs control_Veh and Ang II_Cmpd17b vs AngII_Veh group.*

### *Supplementary Table S23. Functional enrichment of proteins from HCF cells proteomics from Ang II_Veh vs control_Veh and Ang II_Cmpd17b vs AngII_Veh group.*

### *Supplementary Table S24. Total identified proteins of HASM cells proteomics from Ang II_Veh vs control_Veh and Ang II_Cmpd17b vs AngII_Veh group.*

### *Supplementary Table S25. Total identified proteins of HCF cells proteomics from Ang II_Veh vs control_Veh and Ang II_Cmpd17b vs AngII_Veh group.*

1. **REFERENCES**
2. Percie du Sert N, Hurst V, Ahluwalia A, Alam S, Avey MT, Baker M, Browne WJ, Clark A, Cuthill IC, Dirnagl U, Emerson M, Garner P, Holgate ST, Howells DW, Karp NA, Lazic SE, Lidster K, MacCallum CJ, Macleod M, Pearl EJ, Petersen OH, Rawle F, Reynolds P, Rooney K, Sena ES, Silberberg SD, Steckler T, Wurbel H. The ARRIVE guidelines 2.0: Updated guidelines for reporting animal research. *PLoS Biol* 2020;**18**:e3000410.
3. Jackson KL, Marques FZ, Lim K, Davern PJ, Head GA. Circadian differences in the contribution of the brain renin-angiotensin system in genetically hypertensive mice. *Frontiers in Physiology* 2018;**9**:231.
4. Jackson KL, Marques FZ, Lim K, Davern PJ, Head GA. Circadian Differences in the Contribution of the Brain Renin-Angiotensin System in Genetically Hypertensive Mice. *Frontiers in Physiology* 2018;**9**.
5. Moore JP, Vinh A, Tuck KL, Sakkal S, Krishnan SM, Chan CT, Lieu M, Samuel CS, Diep H, Kemp-Harper BK, Tare M, Ricardo SD, Guzik TJ, Sobey CG, Drummond GR. M2 macrophage accumulation in the aortic wall during angiotensin II infusion in mice is associated with fibrosis, elastin loss, and elevated blood pressure. *American Journal of Physiology-Heart and Circulatory Physiology* 2015;**309**:H906-H917.
6. Marshall SA, Qin CX, Jelinic M, O’Sullivan K, Deo M, Walsh J, Li M, Parry LJ, Ritchie RH, Leo CH. The novel small-molecule annexin-a1 mimetic, compound 17b, elicits vasoprotective actions in streptozotocin-induced diabetic mice. *Int J Mol Sci* 2020;**21**:1384.
7. Qin CX, May LT, Li R, Cao N, Rosli S, Deo M, Alexander AE, Horlock D, Bourke JE, Yang YH, Stewart AG, Kaye DM, Du X-J, Sexton PM, Christopoulos A, Gao X-M, Ritchie RH. Small-molecule-biased formyl peptide receptor agonist compound 17b protects against myocardial ischaemia-reperfusion injury in mice. *Nat Commun* 2017;**8**:14232.
8. Jackson K, Head GA, Morris BJ, Chin-Dusting J, Jones E, La Greca L, Mayorov DN. Reduced cardiovascular reactivity to stress but not feeding in renin enhancer knockout mice. *American Journal of Hypertension* 2007;**20**:893-899.
9. Head GA, Lukoshkova EV, Burke SL, Malpas SC, Lambert EA, Janssen BJ. Comparing spectral and invasive estimates of baroreflex gain. *IEEE Eng Med Biol Mag* 2001;**20**:43-52.
10. Head GA, Obeyesekere VR, Jones ME, Simpson ER, Krozowski ZS. Aromatase-deficient (ArKO) mice have reduced blood pressure and baroreflex sensitivity. *Endocrinology* 2004;**145**:4286-4291.
11. Schindelin J, Arganda-Carreras I, Frise E, Kaynig V, Longair M, Pietzsch T, Preibisch S, Rueden C, Saalfeld S, Schmid B, Tinevez JY, White DJ, Hartenstein V, Eliceiri K, Tomancak P, Cardona A. Fiji: an open-source platform for biological-image analysis. *Nat Methods* 2012;**9**:676-682.
12. Tate M, Prakoso D, Willis AM, Peng C, Deo M, Qin CX, Walsh JL, Nash DM, Cohen CD, Rofe AK, Sharma A, Kiriazis H, Donner DG, De Haan JB, Watson AMD, De Blasio MJ, Ritchie RH. Characterising an alternative murine model of diabetic cardiomyopathy. *Frontiers in Physiology* 2019;**10**:1395.
13. Chinnakkannu P, Reese C, Gaspar JA, Panneerselvam S, Pleasant-Jenkins D, Mukherjee R, Baicu C, Tourkina E, Hoffman S, Kuppuswamy D. Suppression of angiotensin II-induced pathological changes in heart and kidney by the caveolin-1 scaffolding domain peptide. *Plos One* 2018;**13**:e0207844.
14. Broekmans K, Giesen J, Menges L, Koesling D, Russwurm M. Angiotensin II-induced cardiovascular fibrosis is attenuated by NO-sensitive guanylyl cyclase1. *Cells* 2020;**9**:2436.
15. Wang H, Yus MM, Brady T, Choi R, Nandakumar K, Smith L, Jang R, Wodu BP, Almodiel D, Stoddart L, Kim DH, Steppan J, Santhanam L. Targeting LOXL2 improves arterial stiffness and function in angiotensin ii-induced hypertension in males but not females. *bioRxiv* 2023;**12**:571541.
16. Tani T, Orimo H, Shimizu A, Tsuruoka S. Development of a novel chronic kidney disease mouse model to evaluate the progression of hyperphosphatemia and associated mineral bone disease. *Sci Rep-Uk* 2017;**7**:2233.
17. Sun Y, Byon CH, Yang Y, Bradley WE, Dell'Italia LJ, Sanders PW, Agarwal A, Wu H, Chen Y. Dietary potassium regulates vascular calcification and arterial stiffness. *JCI Insight* 2017;**2**:e94920.
18. Berg S, Kutra D, Kroeger T, Straehle CN, Kausler BX, Haubold C, Schiegg M, Ales J, Beier T, Rudy M, Eren K, Cervantes JI, Xu B, Beuttenmueller F, Wolny A, Zhang C, Koethe U, Hamprecht FA, Kreshuk A. ilastik: interactive machine learning for (bio)image analysis. *Nat Methods* 2019;**16**:1226-1232.
19. Thallas-Bonke V, Tan SM, Lindblom RS, Snelson M, Granata C, Jha JC, Sourris KC, Laskowski A, Watson A, Tauc M, Rubera I, Zheng GP, Shah AM, Harris DCH, Elbatreek MH, Kantharidis P, Cooper ME, Jandeleit-Dahm K, Coughlan MT. Targeted deletion of nicotinamide adenine dinucleotide phosphate oxidase 4 from proximal tubules is dispensable for diabetic kidney disease development. *Nephrol Dial Transpl* 2021;**36**:988-997.
20. Parker AM, Tate M, Prakoso D, Deo M, Willis AM, Nash DM, Donner DG, Crawford S, Kiriazis H, Granata C, Coughlan MT, De Blasio MJ, Ritchie RH. Characterisation of the myocardial mitochondria structural and functional phenotype in a murine model of diabetic cardiomyopathy. *Frontiers in Physiology* 2021;**12**:672252.
21. Chan CT, Sobey CG, Lieu M, Ferens D, Kett MM, Diep H, Kim HA, Krishnan SM, Lewis CV, Salimova E, Tipping P, Vinh A, Samuel CS, Peter K, Guzik TJ, Kyaw TS, Toh BH, Bobik A, Drummond GR. Obligatory role for b cells in the development of angiotensin ii-dependent hypertension. *Hypertension* 2015;**66**:1023-1033.
22. Acin-Perez R, Benador IY, Petcherski A, Veliova M, Benavides GA, Lagarrigue S, Caudal A, Vergnes L, Murphy AN, Karamanlidis G, Tian R, Reue K, Wanagat J, Sacks H, Amati F, Darley-Usmar VM, Liesa M, Divakaruni AS, Stiles L, Shirihai OS. A novel approach to measure mitochondrial respiration in frozen biological samples. *EMBO J* 2020;**39**:e104073.
23. Tham YK, Bernardo BC, Claridge B, Yildiz GS, Woon LM-L, Bond S, Fang H, Ooi JYY, Matsumoto A, Luo J, Tai CMK, Harmawan CA, Kiriazis H, Donner DG, Mellett NA, Abel ED, Khan SA, De Souza DP, Doomun SNE, Liu K, Xiang R, Singh M, Inouye M, Meikle PJ, Weeks KL, Drew BG, Greening DW, McMullen JR. Estrogen receptor alpha deficiency in cardiomyocytes reprograms the heart-derived extracellular vesicle proteome and induces obesity in female mice. *Nature Cardiovascular Research* 2023;**2**:268-289.
24. Lozano J, Rai A, Lees JG, Fang H, Claridge B, Lim SY, Greening DW. Scalable generation of nanovesicles from human-induced pluripotent stem cells for cardiac repair. *Int J Mol Sci* 2022;**23**:14334.
25. Rai A, Fang H, Claridge B, Simpson RJ, Greening DW. Proteomic dissection of large extracellular vesicle surfaceome unravels interactive surface platform. *J Extracell Vesicles* 2021;**10**:e12164.
26. Claridge B, Rai A, Fang H, Matsumoto A, Luo J, McMullen JR, Greening DW. Proteome characterisation of extracellular vesicles isolated from heart. *Proteomics* 2021;**21**:e2100026.
27. Dai F, Qi Y, Guan W, Meng G, Liu Z, Zhang T, Yao W. RhoGDI stability is regulated by SUMOylation and ubiquitination via the AT1 receptor and participates in Ang II-induced smooth muscle proliferation and vascular remodeling. *Atherosclerosis* 2019;**288**:124-136.
28. Zhou C, Lin Z, Cao H, Chen Y, Li J, Zhuang X, Ma D, Ji L, Li W, Xu S, Pan B, Zheng L. Anxa1 in smooth muscle cells protects against acute aortic dissection. *Cardiovasc Res* 2022;**118**:1564-1582.
29. Cross J, Rai A, Fang H, Claridge B, Greening DW. Rapid and in‐depth proteomic profiling of small extracellular vesicles for ultralow samples. *Proteomics* 2023:2300211.
30. Claridge B, Rai A, Lees JG, Fang H, Lim SY, Greening DW. Cardiomyocyte intercellular signalling increases oxidative stress and reprograms the global‐and phospho‐proteome of cardiac fibroblasts. *Journal of Extracellular Biology* 2023;**2**:e125.
31. Tyanova S, Temu T, Cox J. The MaxQuant computational platform for mass spectrometry-based shotgun proteomics. *Nat Protoc* 2016;**11**:2301-2319.
32. Cox J, Neuhauser N, Michalski A, Scheltema RA, Olsen JV, Mann M. Andromeda: a peptide search engine integrated into the MaxQuant environment. *J Proteome Res* 2011;**10**:1794-1805.
33. Evans J, Hutchison J, Salamonsen LA, Greening DW. Proteomic insights into endometrial receptivity and embryo-endometrial epithelium interaction for implantation reveal critical determinants of fertility. *Proteomics* 2020;**20**:e1900250.
34. Demichev V, Messner CB, Vernardis SI, Lilley KS, Ralser M. DIA-NN: neural networks and interference correction enable deep proteome coverage in high throughput. *Nat Methods* 2020;**17**:41-44.
35. Tyanova S, Cox J. Perseus: a bioinformatics platform for integrative analysis of proteomics data in cancer research. *Methods Mol Biol* 2018;**1711**:133-148.
36. Notaras M, Lodhi A, Dundar F, Collier P, Sayles NM, Tilgner H, Greening D, Colak D. Schizophrenia is defined by cell-specific neuropathology and multiple neurodevelopmental mechanisms in patient-derived cerebral organoids. *Mol Psychiatry* 2022;**27**:1416-1434.
37. Singh J, Jackson KL, Tang FS, Fu T, Nowell C, Salimova E, Kiriazis H, Ritchie RH, Head GA, Woodman OL, Qin CX. The pro-resolving mediator, annexin A1 regulates blood pressure, and age-associated changes in cardiovascular function and remodeling. *FASEB J* 2024;**38**:e23457.
